# Application of oral sulfate solution combined with linaclotide in bowel preparation for colonoscopy

**DOI:** 10.3389/fmed.2026.1696298

**Published:** 2026-03-10

**Authors:** Haifeng Lan, Mengjie Lu, Shupei Li, Mei Shao, Ya Yang, Qi Zhai, Qing Gao, Yuxiu Liu, Ji Xuan

**Affiliations:** 1 Department of Gastroenterology, Jinling Clinical Medical College, Nanjing Medical University, Nanjing, Jiangsu, China; 2 Health Science Center, Ningbo University, Ningbo, Zhejiang, China; 3 Department of Gastroenterology, Jinling School of Clinical Medicine, Nanjing University of Chinese Medicine, Nanjing, Jiangsu, China; 4 Department of Gastroenterology, Affiliated Jinling Hospital, Medical School of Nanjing University, Nanjing, Jiangsu, China; 5 Data and Statistics Division of Department of Critical Care Medicine, Affiliated Jinling Hospital, Medical School of Nanjing University, Nanjing, Jiangsu, China

**Keywords:** oral sulfate solution, linaclotide, PEG, bowel preparation, colonoscopy

## Abstract

**Objective:**

High-quality bowel preparation is essential for accurate colonoscopy. Currently, linaclotide is used as an adjunctive agent supporting bowel preparation, but evidence for its combination with oral sulfate solution (OSS) remains limited. This study aimed to evaluate whether the addition of linaclotide to OSS could improve the efficacy of bowel preparation.

**Methods:**

This single-blind, randomized controlled trial enrolled outpatients scheduled for colonoscopy. Participants were randomly assigned (1:1:1) to one of three groups: the OSS + Linaclotide group, the OSS group, or the polyethylene glycol (PEG) group. The primary outcome was the adequate bowel preparation rate, while secondary outcomes included tolerability, colonoscopy findings, and safety. Efficacy endpoints were assessed in the modified intention-to-treat (mITT) population, and safety was evaluated in the as-treated population.

**Results:**

A total of 370 patients were included in the mITT analysis. There was no statistically significant difference in the adequate bowel preparation rate between the OSS + Linaclotide group and the OSS group (86.5% vs. 88.0%; unadjusted relative risk [RR], 0.98; 95% confidence interval [CI], 0.89–1.08; *p* = 0.723). In the unadjusted analysis, both the OSS + Linaclotide group (86.5% vs. 73.9%; unadjusted RR, 1.17; 95% CI, 1.03–1.33; *p* = 0.013) and the OSS group (88.0% vs. 73.9%; unadjusted RR, 1.19; 95% CI, 1.05–1.35; *p* = 0.005) demonstrated significantly higher adequate bowel preparation rates than the PEG group. Moreover, the analysis adjusted for covariates showed no statistically significant differences between groups (*p* > 0.05). In exploratory analysis, patient-reported incidence of bloating was less frequent in the OSS + Linaclotide group (4.0% vs. 16.0% vs. 11.8%, *p* = 0.007), with no increase in nausea, vomiting, or other adverse events. No significant between-group differences were observed in other tolerability measures or colonoscopy findings.

**Conclusion:**

This study did not meet its primary endpoint. Linaclotide added to OSS provided no additional cleansing efficacy over OSS alone or PEG. Exploratory analysis found that the combination may reduce bloating, a potential tolerability advantage requiring further verification in future studies.

## Graphical Abstract

**Figure:**
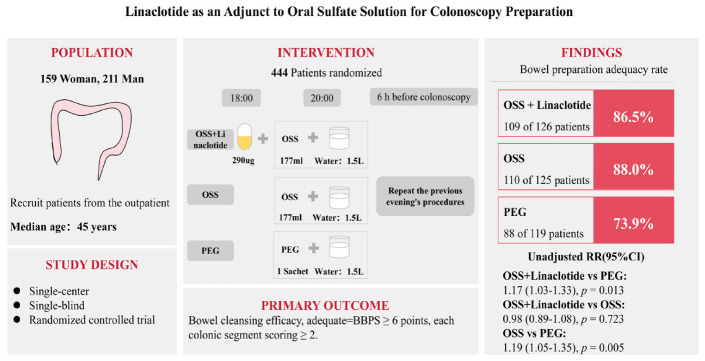
Linaclotide as an adjunct to oral sulfate solution for colonoscopy preparation.

## Introduction

Colonoscopy is the most commonly used and intuitive diagnostic method for intestinal diseases, with its diagnostic quality closely related to the effectiveness of bowel preparation (1). Currently, numerous bowel cleansing agents are available both domestically and internationally, each with unique advantages and limitations. Consequently, optimizing bowel preparation remains a significant clinical challenge and a persistent research focus.

Polyethylene glycol (PEG), the most widely used bowel cleansing agent, has been extensively recognized for its safety profile (2) and is recommended by the European Society of Gastrointestinal Endoscopy (ESGE) (3). The 3L PEG regimen has now become the standard control protocol in clinical studies. However, its suboptimal palatability frequently compromises patient adherence, necessitating ongoing efforts to optimize the formulation and sensory properties of bowel cleansing agents (4).

Oral sulfate solution (OSS) is a newly marketed modified osmotic laxative with an orange flavor and improved palatability. The formulation contains three sulfate salts (sodium, magnesium, and potassium sulfates), which reduce the risk of fluid and electrolyte disturbances during bowel cleansing (5). Phase III clinical trials have shown that split-dose OSS is non-inferior to 3L PEG in efficacy, with comparable safety and acceptability profiles between the two groups (6). Compared to 2L PEG/ascorbic acid (PEG/Asc), OSS demonstrated similar efficacy and tolerability, underscoring its effectiveness as a bowel preparation agent (7).

Linaclotide is a guanylate cyclase-C (GC-C) receptor agonist, FDA-approved for the treatment of constipation-predominant irritable bowel syndrome (IBS-C) (8). Studies have shown that linaclotide combined with 3L PEG significantly improves bowel preparation quality in the right colon, shortens the time to first bowel movement, and enhances patient satisfaction (9), indicating its potential as an adjunctive agent for bowel preparation.

At present, there are relatively few studies on the combination of OSS and linaclotide for bowel preparation. This study conducted a single-center, single-blind, randomized controlled clinical trial, aiming to explore the efficacy and safety of OSS + Linaclotide compared with OSS alone and PEG in bowel preparation.

## Materials and methods

### Study design

This study was an investigator-initiated, single-center, randomized trial conducted at Affiliated Jinling Hospital, Medical School of Nanjing University, from September 2023 to February 2024, with blinded assessment of primary outcomes. All participants provided written informed consent after being fully informed. The trial was conducted in strict accordance with the principles of the Declaration of Helsinki. It received approval from the institutional medical ethics committee (approval number: DZQH-KYLL-23-11) and was registered on ClinicalTrials.gov (registration number: NCT06091735). The trial protocol provided detailed information on the trial site, a list of investigators and administrators, drug details, and a flowchart of the trial design (Supplementary Material 2).

### Patients

This trial enrolled participants aged 18–80 years, with no gender restriction, who were scheduled for colonoscopy and able to adhere to the study procedures, follow-up schedule, and complete the required questionnaires. Exclusion criteria included: (1) severe cardiopulmonary, hepatic, or renal dysfunction; (2) suspected gastrointestinal obstruction or perforation; (3) pregnant or lactating women, or those planning pregnancy during the trial period; (4) mental illness or physical disability preventing cooperation with the examination; (5) allergy to bowel preparation medications; (6) failure to undergo colonoscopy after bowel cleansing; (7) withdrawal due to inability to tolerate standard colonoscopy; (8) voluntary withdrawal from the study; (9) participation in other clinical trials within 3 months prior to or during the trial. Eligibility criteria are detailed in the trial protocol (Supplementary Material 2).

### Randomization

Eligible patients were randomly assigned in a 1:1:1 ratio to the OSS + Linaclotide group, OSS group, or PEG group, with 148 participants per group. All participants completed enrollment allocation in an outpatient setting, followed by scheduling of colonoscopy appointments. A fixed block randomization scheme with a block size of 6 was employed. Once patient eligibility was confirmed, randomization was immediately carried out via a secure randomization website. Subsequently, clinical staff assigned interventions, while endoscopists remained blinded to the intervention allocation until the study was completed and the database was locked.

### Intervention

All participants received face-to-face instruction from the research team, covering medication administration methods, dosages, timing, management of adverse reactions, and potential consequences of inadequate bowel preparation. Reference videos were provided to aid comprehension. The detailed medication regimen was as follows: on the evening before colonoscopy, participants consumed dinner before 18:00. At 20:00, they began taking the bowel cleansing agent dissolved in water, followed by approximately 500 mL of water consumed evenly over the next 2 h (total fluid intake: 1.5 L). Six hours prior to the scheduled colonoscopy time, participants ingested the second dose of the bowel cleansing agent and one 20 mL bottle of simethicone emulsion, followed by 1.5 L of consumed within 2 h. Immediately before colonoscopy, an additional 20 mL bottle of simethicone was administered. For the OSS + Linaclotide group, participants first orally took one 290 μg linaclotide capsule at 18:00 on the evening prior to colonoscopy, with subsequent steps identical to the other groups. All groups adhered to the “6 + 2” protocol: strict fasting for 6 h and strict fluid restriction for 2 h prior to colonoscopy (10).

### Outcomes

The primary outcome was bowel cleansing efficacy. It was assessed using the Boston Bowel Preparation Scale (BBPS) to compare the total scores and segmental scores across the three groups. Adequate bowel preparation was defined as a total BBPS score ≥ 6, with a score of ≥2 in each colonic segment. The adequate preparation rate was calculated as (the number of participants meeting the criteria/the total number of participants) × 100%. The detailed BBPS scoring criteria were specified in the clinical study protocol (Supplementary Material 2) (11, 12).

Secondary outcomes included bowel bubble score (BBS, 0-3 score) (12), patient-reported experience (taste satisfaction [best/worse], sleep quality [best/same as before/worse], satisfaction with educational methods [yes/no], willingness to repeat [yes/no], and adherence [yes/no]), as well as colonoscopy findings. Colonoscopy findings comprised polyp detection rate (proportion of participants with at least one detected polyp among all participants), adenoma detection rate, and carcinoma detection rate (proportion of participants with at least one pathologically confirmed adenoma or carcinoma among total participants). Safety was assessed by recording the presence or absence of predefined adverse events, including nausea, vomiting, bloating, and abdominal pain.

All participants were required to complete a questionnaire prior to colonoscopy. Bowel preparation efficacy was then objectively assessed during colonoscopy by 12 endoscopists (each with ≥5 years of experience and >2000 independent procedures) using the BBPS and BBS. During the procedure, the colonoscopy withdrawal time was at least 6 min (excluding time required for biopsy sampling or lesion resection).

### Statistical analysis

The primary objective of this study was to compare the efficacy between the group OSS + Linaclotide and the PEG group. Based on literature data from similar study designs (3, 13, 14), we set the expected adequate bowel preparation rates for the OSS + Linaclotide group, OSS monotherapy group, and PEG group at 90%, 86%, and 69.36%, respectively. A two-sided significance level of α = 0.05 and a statistical power of 80% were established, with samples allocated in a 1:1:1 ratio across the three groups. The minimum required sample size per group was calculated as 118 participants. Accounting for a 20% dropout rate, the planned sample size per group was at least 148 participants.

The intention-to-treat (ITT) population comprised all randomized participants. The primary efficacy analysis was conducted in a modified intention-to-treat (mITT) population, defined as those who initiated the assigned bowel preparation regimen, excluding participants who did not take the study medication or undergo colonoscopy after randomization. This mITT definition was used as bowel preparation quality can only be assessed upon colonoscopy completion. The per-protocol (PP) population, consisting of participants who fully adhered to the protocol by completing the bowel preparation regimen and undergoing colonoscopy, was also analyzed. The primary outcome, namely adequate bowel preparation, was analyzed using a modified Poisson regression model adjusted for prespecified covariates (gender, age, drinking, smoking, history of abdominal surgery, and first colonoscopy examination). This analysis yielded adjusted risk ratios (RRs) along with their corresponding 95% confidence intervals (CIs) as the measure of treatment effect. To evaluate the robustness of the primary finding with respect to potential attrition bias, worst-case and best-case scenario sensitivity analyses were carried out. Analyses of serious adverse events and related complications were performed in the safety population, which comprised all randomized participants who received the intervention. Secondary and safety outcomes were analyzed without covariate adjustment. All *p*-values are presented without adjustment for multiple comparisons and were considered statistically significant at *p* < 0.05.

This trial did not perform imputation for missing data, as the datasets used for the primary analysis and prespecified covariate-adjusted analyses were complete. Data cleaning and statistical analyses were conducted using R version 4.5.0. Additional supplementary information on statistical analyses is provided in Supplementary Material 3.

## Results

### Baseline characteristics

A total of 370 participants were included in the mITT analysis (126 in the OSS + Linaclotide group, 125 in the OSS group, and 119 in the PEG group). Additionally, 363 participants were included in the PP analysis, with 126 in the OSS + Linaclotide group, 123 in the OSS group, and 114 in the PEG group (Supplementary Figure 1). The median age of the patients was 45 years (interquartile range, 34–60 years), and 211 patients (57.0%) were male. Among all participants, 230 (62.3%) underwent colonoscopy for the first time. The indications for colonoscopy included screening for bowel diseases in 163 patients (44.0%), diagnostic examinations in 135 patients (36.5%), and follow-up or surveillance in 72 patients (19.5%). The demographic and clinical characteristics were well-balanced across the three groups (Table 1 and Supplementary Table 1).

**TABLE 1:** Baseline characteristics (modified intention-to-treat population).

Characteristics	OSS + Linaclotide (*n* = 126)	OSS group (*n* = 125)	PEG group (*n* = 119)
Age, yr	45.0 (35.0–60.0)	43.5 (34.0–61.3)	46.5 (33.0–60.0)
**Sex**			
Male	69 (54.8)	76 (60.8)	66 (55.5)
Female	57 (45.2)	49 (39.2)	53 (44.5)
BMI, kg/m^2^	23.7 (21.6–26.1)	24.0 (21.6–25.6)	23.7 (21.6–26.1)
**Medical history**			
Drinking	29 (23.0)	34 (27.2)	38 (31.9)
Smoking	31 (24.6)	30 (24.0)	30 (25.2)
Hypertension	20 (15.9)	18 (14.4)	20 (16.8)
Diabetes	5 (4.0)	6 (4.8)	4 (3.4)
History of abdominal surgery	26 (20.6)	24 (19.2)	24 (20.2)
History of polyp resection	22 (17.5)	19 (15.2)	16 (13.4)
Constipation	11 (8.7)	10 (8.0)	14 (11.8)
Others	17 (13.5)	18 (14.4)	23 (19.3)
**First colonoscopy examination**			
Yes	82 (65.1)	74 (59.2)	74 (62.2)
No	44 (34.9)	51 (40.8)	45 (37.8)
Indication of colonoscopy*			
Screening	58 (46.0)	53 (42.4)	52 (43.7)
Diagnosis	43 (34.1)	44 (35.2)	48 (40.3)
Surveillance	25 (19.8)	28 (22.4)	19 (16.0)

OSS + Linaclotide Group, oral sulfate solution + 290 ug Linaclotide (guanylate cyclase-C agonist, promoting intestinal secretion); OSS, oral sulfate solution alone; PEG, polyethylene glycol; BMI, body mass index. *Screening: Screening or Physical Examination. This mainly includes individuals requesting colonoscopy due to reasons such as elevated tumor markers, esophagitis, gastritis, gastric ulcer, and other gastric symptoms. Diagnosis: Diagnosis with Definite Symptoms. This includes abdominal symptoms such as diarrhea, abdominal pain, occult blood or blood in stool, weight loss, etc. Surveillance: Follow-up or Re-examination. This applies to individuals with a history of enteritis, intestinal polyps, tumors, surgery, or hemorrhoids. Data are presented as [Median (IQR)] or *n* (%).

### Primary outcome

**FIGURE 1:**
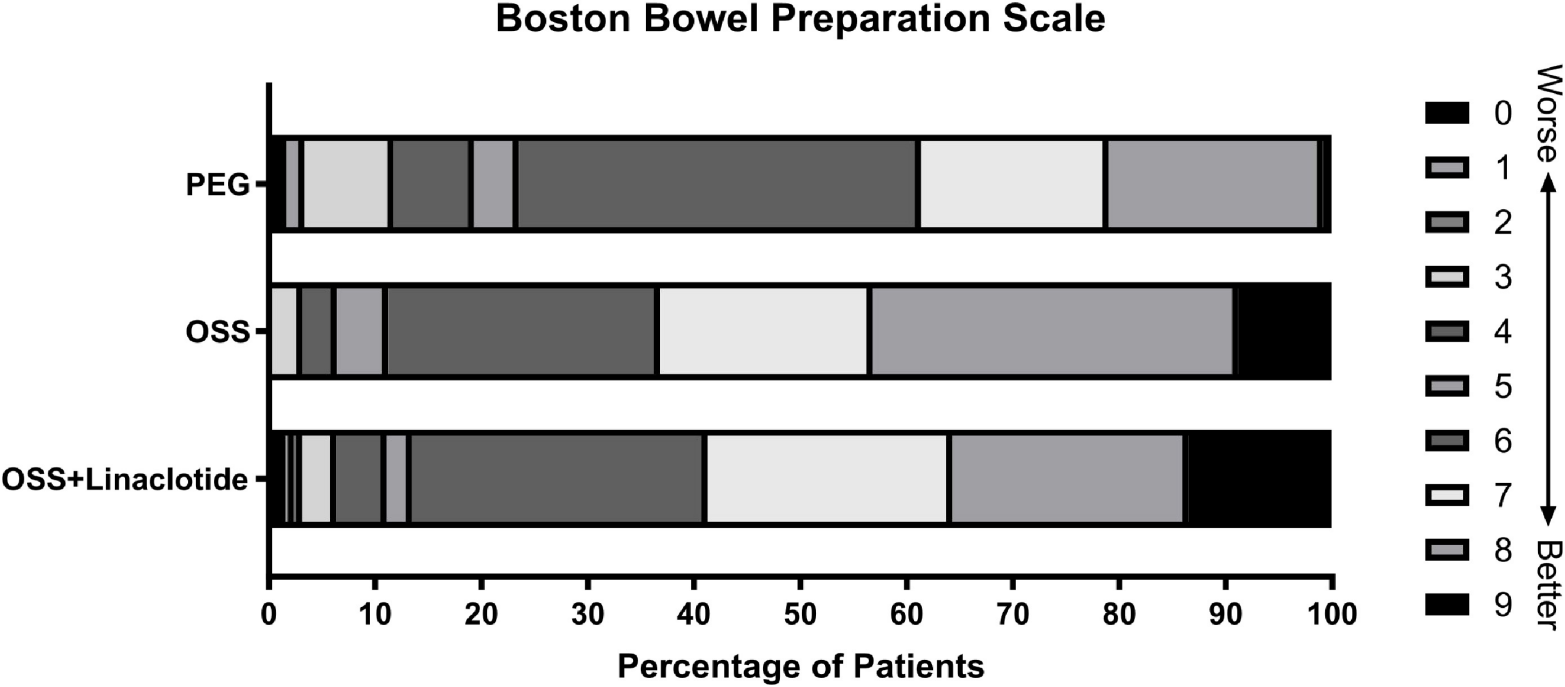
Boston Bowel Preparation Scale score distribution in the modified intention-to-treat population. Shown is the distribution of scores on the Boston Bowel Preparation Scale (BBPS) in the three intervention groups. Scores ranged from 0 to 9, with 0 indicating the poorest bowel preparation, ≥6 denoting adequate preparation (pass threshold), and 9 representing optimal preparation. Higher scores correlate with better bowel cleansing quality. For comprehensive assessment of bowel preparation efficacy, this figure must be analyzed in conjunction with segment-specific BBPS scores (right colon, transverse colon, left colon), each rated on a 0–3 scale. The primary outcome was defined as bowel preparation adequacy rate, with adequacy criteria set as total BBPS ≥ 6 and individual segment scores ≥2 for all colonic regions.

**FIGURE 2:**
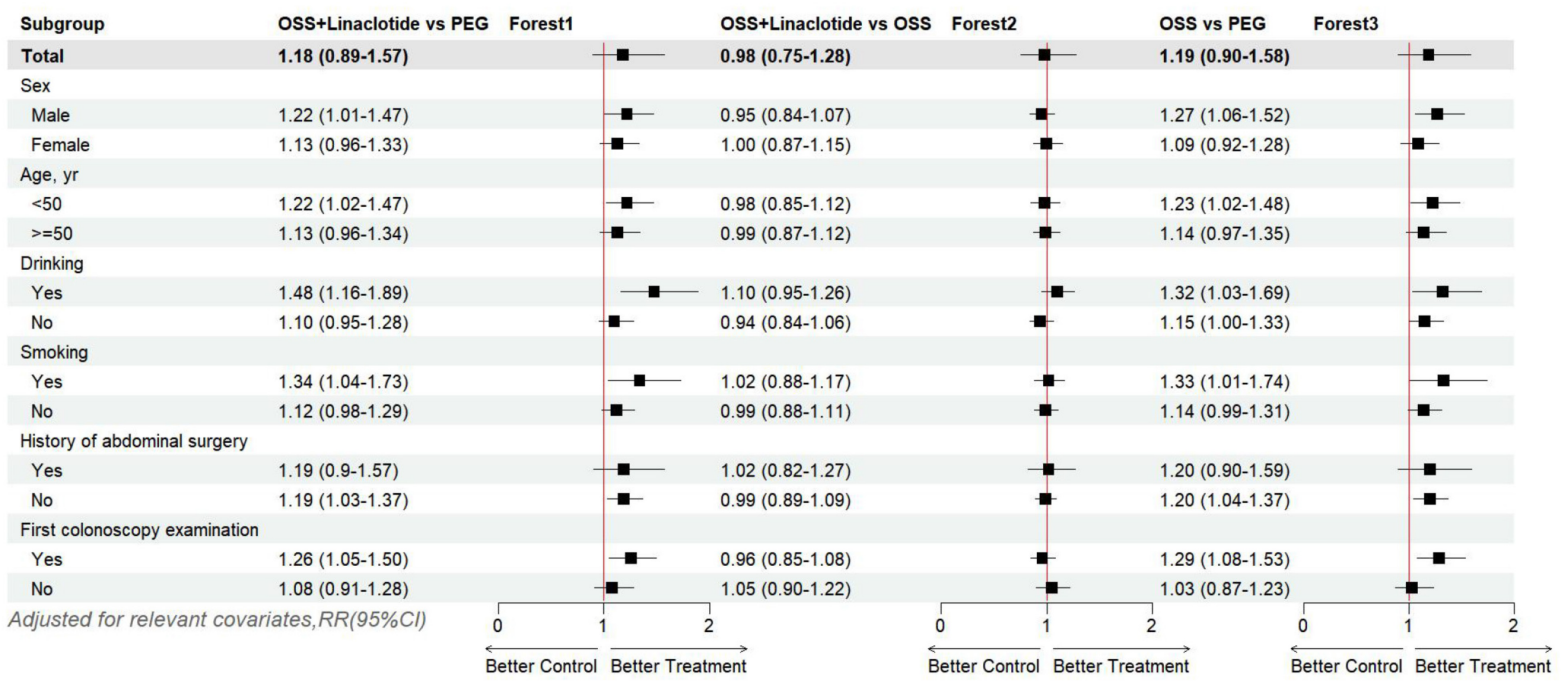
Subgroup analysis of bowel preparation quality (modified intention-to-treat population). The forest plot shows the adjusted risk ratio (RR) and 95% CI between the three intervention groups for bowel preparation quality, adjusted risk ratios are reported in accordance with the prespecified statistical analysis plan. The widths of the confidence intervals were adjusted for multiplicity. For populations with a history of smoking, alcohol, or abdominal surgery (*n* < 30), the results should be interpreted with caution.

In the mITT population, no statistically significant difference was observed in the adequate bowel preparation rate between the OSS + Linaclotide group and the OSS group (86.5% vs. 88.0%; unadjusted RR: 0.98; 95% CI: 0.89–1.08; *p* = 0.723). In unadjusted analysis, both the OSS + Linaclotide group (86.5% vs. 73.9%; unadjusted RR: 1.17; 95% CI: 1.03–1.33; *p* = 0.013) and the OSS group (88.0% vs. 73.9%; unadjusted RR: 1.19; 95% CI: 1.05–1.35; *p* = 0.005) demonstrated significantly higher adequate bowel preparation rate than the PEG group. Moreover, the analysis adjusted for covariates showed no statistically significant differences between groups (*p* > 0.05) (Table 2). Sensitivity analyses were conducted to assess the potential impact of missing data from 74 excluded participants. Under both the best-case (88.5% vs. 89.9% vs. 79.1%, *p* = 0.015) and worst-case analysis (73.6% vs. 74.3% vs. 59.5%, *p* = 0.008), the results remained consistent with those of the primary analysis (Supplementary Table 7). No differences in BBPS total scores or segmental scores were found between the OSS + Linaclotide group and the OSS group (*p* > 0.05), while both groups significantly outperformed the PEG group (*p* < 0.05) (Supplementary Table 2 and Figure 1). The results in the PP population were consistent with those in the mITT population. Subgroup analyses of bowel preparation efficacy adjusted for covariates are presented in Figure 2, with unadjusted analyses in Supplementary Figure 2.

**TABLE 2:** Bowel preparation quality comparison.

Comparison	Bowel preparation adequacy rate comparison	Unadjusted RR (95% CI)	*p*	Adjusted RR (95% CI)	*p*
Modified intention-to-treat population					
OSS + Linaclotide vs. PEG group	86.5% vs. 73.9%	1.17 (1.03–1.33)	0.013	1.18 (0.89–1.57)	0.241
OSS + Linaclotide vs. OSS group	86.5% vs. 88.0%	0.98 (0.89–1.08)	0.723	0.98 (0.75–1.28)	0.891
OSS vs. PEG group	88.0% vs. 73.9%	1.19 (1.05–1.35)	0.005	1.19 (0.90–1.58)	0.224
Per-protocol analysis					
OSS + Linaclotide vs. PEG group	86.5% vs. 75.4%	1.15 (1.01–1.30)	0.028	1.16 (0.87–1.55)	0.304
OSS + Linaclotide vs. OSS group	86.5% vs. 87.8%	0.98 (0.90–1.08)	0.760	0.98 (0.75–1.29)	0.911
OSS vs. PEG group	87.8% vs. 75.4%	1.16 (1.03–1.32)	0.014	1.17 (0.88–1.55)	0.287

OSS + Linaclotide group, oral sulfate solution + 290 ug linaclotide (guanylate cyclase-C agonist, promoting intestinal secretion); OSS, oral sulfate solution alone; PEG, polyethylene glycol; Pairwise comparisons of bowel preparation adequate rates were conducted among the three intervention groups. Covariate adjustments were performed for gender, age, alcohol consumption, smoking, history of abdominal surgery, and whether the colonoscopy was the first-time procedure. The table presented both unadjusted and adjusted risk ratios (RR) with 95% confidence intervals (CI).

### Secondary outcomes

No significant differences were observed among the three groups in terms of bowel bubble scores (Supplementary Table 6) or tolerance (including satisfaction with taste, satisfaction with educational methods, sleep quality, and willingness to repeat the regimen) (Supplementary Table 3). Similarly, there were no significant differences in polyp detection rates (43.7% vs. 45.5% vs. 42.1%, *p* = 0.868), adenoma detection rates (18.3% vs. 21.1% vs. 15.8%, *p* = 0.568), or cancer detection rates (0.8% vs. 0.8% vs. 1.8%, *p* = 0.740) among the three groups (Supplementary Table 4).

### Safety outcomes

**FIGURE 3:**
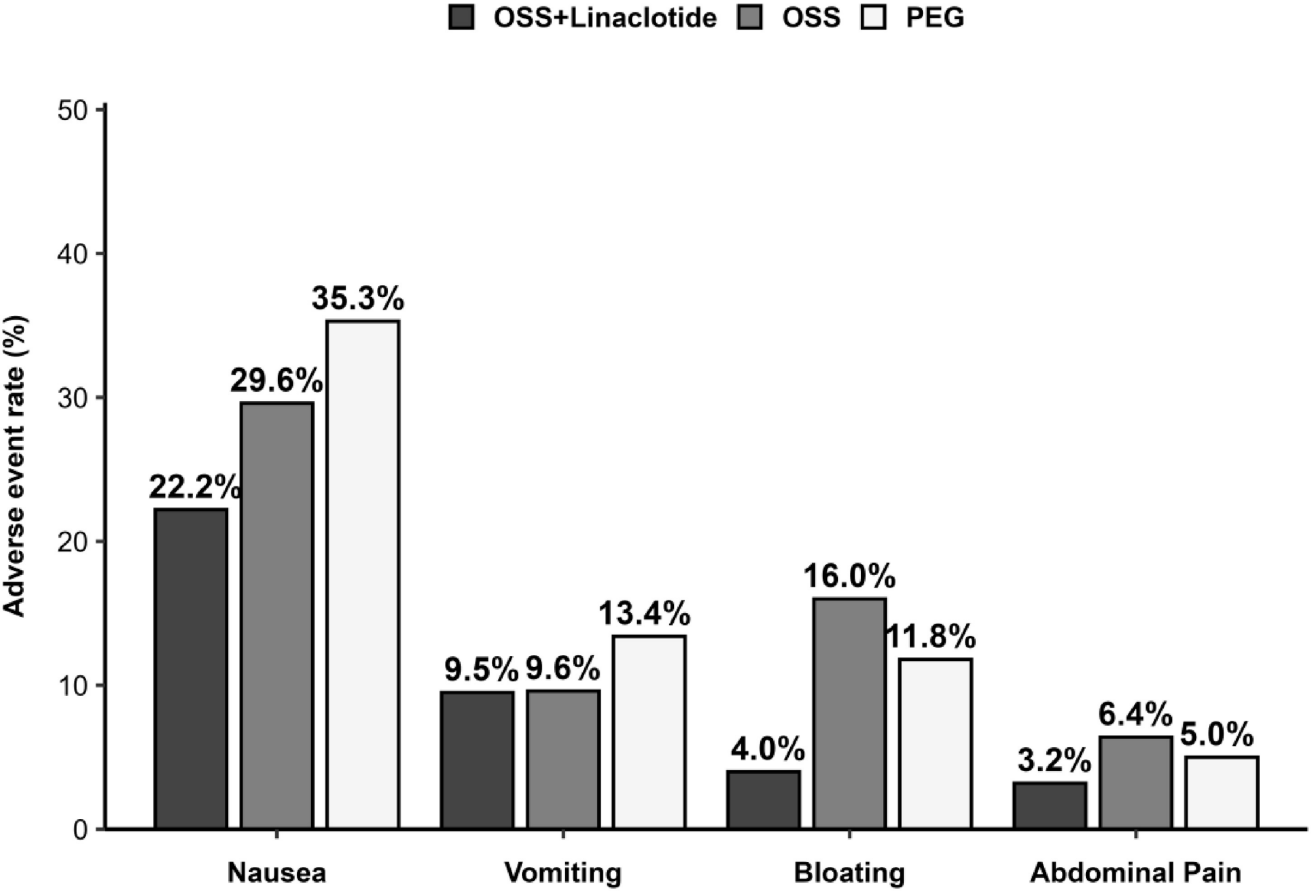
Adverse events (modified intention-to-treat population).

The OSS + Linaclotide group was associated with significantly lower rates of overall adverse events compared to the OSS alone and PEG groups (35.7% vs. 47.2% vs. 50.4%, *p* = 0.05) and a significantly lower incidence of bloating (4.0% vs. 16.0% vs. 11.8%, *p* = 0.007) (Supplementary Table 5). The incidence rates of nausea, vomiting, and abdominal pain did not differ significantly among the three groups (Figure 3). No severe adverse events were reported in any group, with all adverse events limited to mild gastrointestinal symptoms.

## Discussion

Early detection, diagnosis, and treatment of intestinal diseases depend on high-quality bowel preparation (15). This randomized trial did not meet its pre-specified primary endpoint. Although an unadjusted analysis showed a significant benefit of OSS + linaclotide over 3L PEG, this difference was no longer statistically significant after adjustment for covariates using the pre-specified modified Poisson model, suggesting that known confounders may have obscured a true treatment effect. Furthermore, linaclotide provided no additive cleansing benefit over OSS alone. This lack of enhanced efficacy may be related to the administration methods, timing, dosage of linaclotide, and potential drug-drug interactions. Previous studies explored various dosing strategies, including single or split doses on the day before colonoscopy, 3-days consecutive administration, or adjusting drug doses (290, 580, 870 μg), among others (16–18). However, an optimal combined regimen has not yet been established, and further randomized controlled trials (RCTs) are needed for exploration. The results in the PP population were consistent with those in the mITT population in terms of overall trends.

The adequate bowel preparation rate in the OSS + Linaclotide group was 86.5%, which did not meet the minimum threshold of >90% recommended by the European Society of Gastrointestinal Endoscopy (ESGE) and the US Multi-Society Task Force on Colorectal Cancer (19). This may be partly attributed to the stringent evaluation criteria, namely a BBPS score ≥ 6 with each segment scoring ≥2. Age, sex, smoking, constipation, and the history of abdominal surgery are currently recognized as independent risk factors for inadequate bowel preparation (20–23). Therefore, these factors were included as prespecified covariates in the adjusted analysis and were examined in subgroup analyses. The subgroup analysis results showed that the OSS regimens (with or without linaclotide) had adjusted RRs > 1 in most subgroups, suggesting that these populations may benefit more from the OSS regimens, which were also significantly superior to PEG. The efficacy of OSS alone was comparable to that of OSS + Linaclotide, as the adjusted RRs for OSS + Linaclotide vs. OSS alone were all close to 1 across all subgroups, supporting the conclusion that there is no difference in efficacy between the two OSS regimens. The PEG regimen performed worse in some subgroups, particularly among drinkers, smokers, patients without a history of abdominal surgery, and first colonoscopy examination. However, the statistical power for analyses of some subgroups (history of smoking, alcohol, or abdominal surgery; *n* < 30) was limited due to small sample sizes, potentially affecting the stability and reliability of these specific findings. Consequently, these subgroup results require validation in large, more balanced cohorts and should be interpreted cautiously. For patients with these characteristics, implementing a tailored bowel preparation regimen may help enhance bowel cleansing efficacy.

Notably, the OSS + Linaclotide group reported a lower incidence of bloating, which may be attributed to its ability to increase small intestinal fluid secretion, accelerate colonic transit, enhance stool water content, and promote defecation, thereby alleviating overall symptoms such as abdominal discomfort, pain, and bloating (16, 24, 25). However, this single-blind design, in which patients were aware of their assigned regimen, introduces a potential risk of performance and reporting bias, especially for subjective symptoms like bloating. Future studies of adjunctive agents like linaclotide should employ a double-blind, placebo-controlled design to reliably assess their impact on tolerance.

## Limitations

This study has several limitations. First, the single-center cohort of relatively young outpatients may limit generalizability to older or higher-risk populations. Second, the single-blind design without placebo control for linaclotide, combined with the use of non-validated instruments for subjective outcomes, introduces potential bias in tolerability assessments. Third, our primary mITT analysis excluded 74 patients (16.7%) who did not initiate the regimen or complete colonoscopy; this non-random attrition may lead to selection bias. Finally, secondary indicators such as satisfaction and tolerance were evaluated using simple, non-validated measures rather than standardized scales, not adjusted for multiple comparisons, and several subgroup analyses were underpowered; thus, these results are exploratory and require cautious interpretation.

## Conclusion

This study did not meet its prespecified primary endpoint. The addition of linaclotide to OSS demonstrated no significant improvement in cleansing efficacy over OSS alone or PEG. Its potential advantage may lie in a reduction in bloating, but this is an exploratory finding that requires verification in rigorously designed trials.

## Data Availability

The raw data supporting the conclusions of this article will be made available by the authors, without undue reservation.
