# Correction: Application of oral sulfate solution combined with linaclotide in bowel preparation for colonoscopy

**DOI:** 10.3389/fmed.2026.1903499

**Published:** 2026-07-02

**Authors:** Haifeng Lan, Mengjie Lu, Shupei Li, Mei Shao, Ya Yang, Qi Zhai, Qing Gao, Yuxiu Liu, Ji Xuan

**Affiliations:** 1 Department of Gastroenterology, Jinling Clinical Medical College, Nanjing Medical University, Nanjing, Jiangsu, China; 2 Health Science Center, Ningbo University, Ningbo, Zhejiang, China; 3 Department of Gastroenterology, Jinling School of Clinical Medicine, Nanjing University of Chinese Medicine, Nanjing, Jiangsu, China; 4 Department of Gastroenterology, Affiliated Jinling Hospital, Medical School of Nanjing University, Nanjing, Jiangsu, China; 5 Data and Statistics Division of Department of Critical Care Medicine, Affiliated Jinling Hospital, Medical School of Nanjing University, Nanjing, Jiangsu, China

**Keywords:** oral sulfate solution, linaclotide, PEG, bowel preparation, colonoscopy

There was a mistake in Table 2 as published. In the original Table 2, the confidence intervals for adjusted risk ratios were calculated using the Wald method, and the *P*-values were derived from Wald tests. Given our limited sample size, binary outcomes, and adjustment for multiple covariates, this approximation was imprecise, leading to logical inconsistencies in the Adjusted RR (95% CI) and *P*-value columns (e.g., confidence intervals that excluded 1 but *P*-values > 0.05). The corrected Table 2 appears below.

**Table 2:** Bowel preparation quality comparison.

Comparison	Bowel preparation adequacy rate comparison	Unadjusted RR (95%CI)	*p*	Adjusted RR (95%CI)	*p*
Modified intention-to-treat population					
OSS + Linaclotide vs. PEG group	86.5% vs. 73.9%	1.17 (1.03–1.33)	0.013	1.18 (0.89–1.57)	0.241
OSS + Linaclotide vs. OSS group	86.5% vs. 88.0%	0.98 (0.89–1.08)	0.723	0.98 (0.75–1.28)	0.891
OSS vs. PEG group	88.0% vs. 73.9%	1.19 (1.05–1.35)	0.005	1.19 (0.90–1.58)	0.224
Per-protocol analysis			
OSS + Linaclotide vs. PEG group	86.5% vs. 75.4%	1.15 (1.01–1.30)	0.028	1.16 (0.87–1.55)	0.304
OSS + Linaclotide vs. OSS group	86.5% vs. 87.8%	0.98 (0.90–1.08)	0.760	0.98 (0.75–1.29)	0.911
OSS vs. PEG group	87.8% vs. 75.4%	1.16 (1.03–1.32)	0.014	1.17 (0.88–1.55)	0.287

OSS + Linaclotide group, oral sulfate solution + 290 ug linaclotide (guanylate cyclase-C agonist, promoting intestinal secretion); OSS, oral sulfate solution alone; PEG, polyethylene glycol; Pairwise comparisons of bowel preparation adequate rates were conducted among the three intervention groups. Covariate adjustments were performed for gender, age, alcohol consumption, smoking, history of abdominal surgery, and whether the colonoscopy was the first-time procedure. The table presented both unadjusted and adjusted risk ratios (RR) with 95% confidence intervals (CI).

Consequently, Figure 2 has also been corrected because its first row of data was derived from Table 2. The corrected Figure 2 appears below.

**Figure 2:**
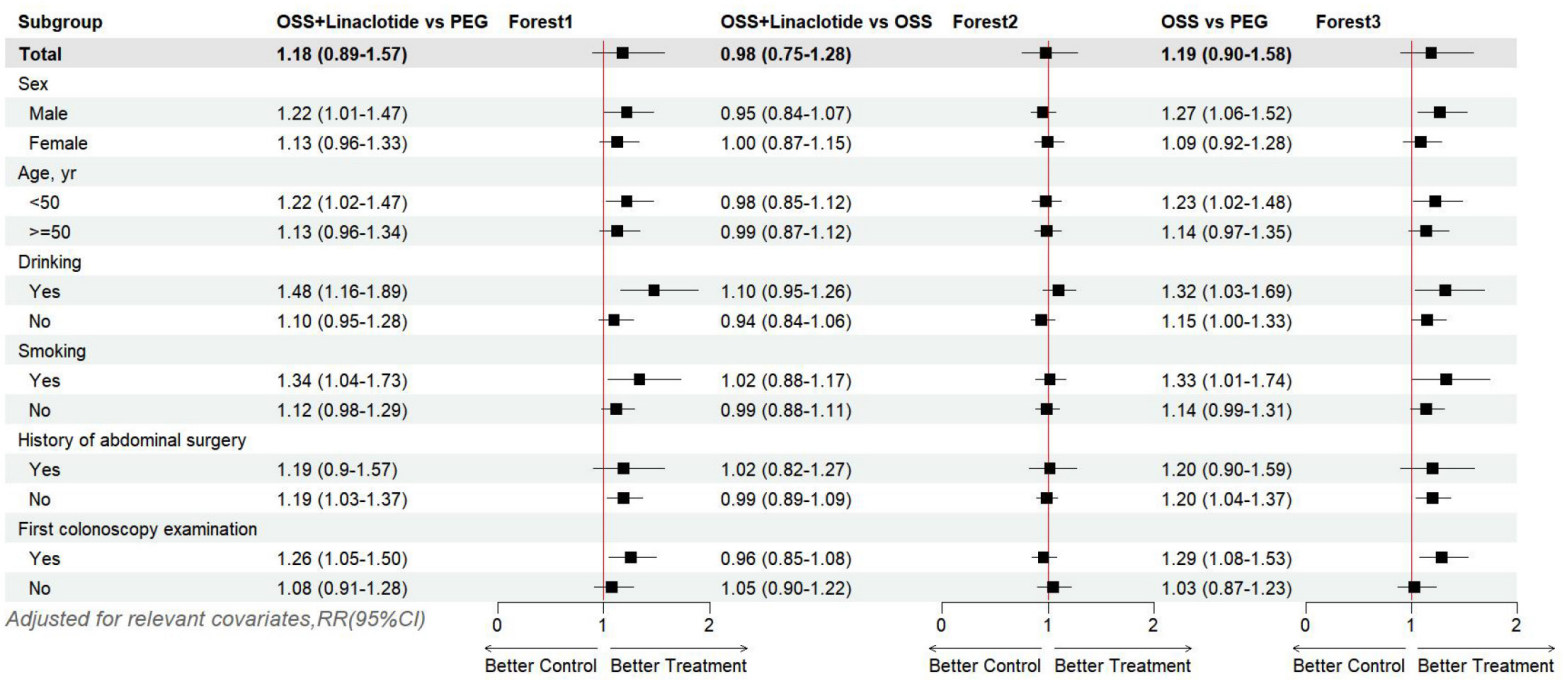
Subgroup analysis of bowel preparation quality (modified intention-to-treat population). The forest plot shows the adjusted risk ratio (RR) and 95% CI between the three intervention groups for bowel preparation quality, adjusted risk ratios are reported in accordance with the pre-specified statistical analysis plan. The widths of the confidence intervals were adjusted for multiplicity. For populations with a history of smoking, alcohol, or abdominal surgery (*n* < 30), the results should be interpreted with caution.

The original version of this article has been updated.

