# Supplementary material for: Application of oral sulfate solution combined with linaclotide in bowel preparation for colonoscopy
Source: Front Med (Lausanne). 2026 Mar 10;13:1696298. doi: 10.3389/fmed.2026.1696298 (PMC13014616; doi:10.3389/fmed.2026.1696298)
Supplement: Supplementary file 1 [file Supplementary_file_1.docx]

**Supplement online content**

**Title：Application of Oral Sulfate Solution Combined with Linaclotide in Bowel Preparation for Colonoscopy**

**Abbreviations**

**Method 1: Drugs information**

**Method 2: Analyze the definition of population**

**Method 3: Supplementary statistical methods**

**Baseline characteristics: Research procedure**

**Supplement Table 1：Baseline characteristics (per-protocol population).**

**Supplement Table 2:Three groups Boston Bowel Preparation Scale score.**

**Supplement Table 3: Tolerance assessment (modified intention-to-treat population).**

**Supplement Table 4: Colonoscopy findings (per-protocol population).**

**Supplement Table 5：Adverse events (modified intention-to-treat population).**

**Supplement Table 6: Three Groups Bowel Bubble Score (per-protocol population).**

**Supplement Table 7: Sensitivity analyses of adequate bowel preparation rate.**

**Supplement Figure 1：Flowchart of the study**

**Supplement Figure 2：Subgroup analysis of bowel preparation quality (modified intent-to-treat population).**

- **Abbreviations**

| **Abbreviations /acronyms** | **Definitions** |
| --- | --- |
| ADR | Adenoma Detection Rate |
| BBPS | Boston Bowel Preparation Scale |
| BBS | Bowel Bubble Score |
| BMI | Body Mass Index |
| BP | Blood Pressure |
| CI | Confidence Interval |
| ESGE | European Society of Gastrointestinal Endoscopy |
| GC-C | Guanylate Cyclase-C |
| IBS | Irritable Bowel Syndrome |
| ITT | Intention-to-Treat |
| mITT | Modified Intention-to-Treat |
| OSS | Oral Sulfate Solution |
| PDR | Polyp Detection Rate |
| PEG | Polyethylene Glycol |
| PP | Per-Protocol |
| RCT | Randomized Controlled Trial |
| RR | Risk Ratio |

- **Method 1: Drugs information**

The Oral Sulfate Solution (OSS), manufactured by Jichuan Pharmaceutical Group Co., Ltd. (specification: 177 mL/bottle; packaged as 2 bottles per box), contains 17.5 g sodium sulfate, 3.13 g potassium sulfate, and 1.6 g magnesium sulfate per 177 mL bottle. Linaclotide (produced by Almac; available as 290-microgram capsules) is also used. Polyethylene glycol (PEG), supplied by Shenzhen Wanhe Pharmaceutical Co., Ltd. (specification: 68.56 g/bag), consists of 1.46 g sodium chloride, 5.68 g anhydrous sodium sulfate, 0.74 g potassium chloride, 1.68 g sodium bicarbonate, and 59 g polyethylene glycol 4000 (PEG-4000) per bag.

- **Method 2: Analyze the definition of population**
- **Intention-to-treat(ITT) population**

The intention-to-treat (ITT) population included all participants who were randomized, irrespective of their subsequent adherence to the study protocol, completion of the bowel preparation regimen, or attendance at colonoscopy.

- **Modified intention-to-treat(mITT) population**

The modified intention-to-treat (mITT) population was a subset of the ITT population, which excluded participants who did not initiate the assigned bowel preparation regimen or who did not present for colonoscopy after randomization.

- **Per-protocol (PP) Population**

The per-protocol (PP) population was a further subset of the mITT population, comprising only participants who fully adhered to the study protocol. This included completing the assigned bowel preparation regimen as prescribed and successfully undergoing colonoscopy.

- **Safety Population**

The Safety Population included all patients who received any dose of the study drug. In cases of protocol violations, patients were classified based on the treatment actually received. Those who withdrew informed consent immediately post-randomization and received no intervention were excluded from the Safety Population. Notably, the Safety Population in this study was identical to the mITT population.

- **Method 3: Supplementary statistical methods**

For normally distributed or approximately normally distributed data, results are presented as mean ± standard deviation, and between-group comparisons were performed using one-way ANOVA followed by LSD post-hoc tests. For skewed data, median (interquartile range) was used for description, and comparisons were conducted via the Kruskal-Wallis test. Categorical data are expressed as frequencies (percentages) [n (%)], with comparisons of rates or proportions performed using the chi-square test or Fisher's exact test. A two-sided P-value <0.05 was considered statistically significant.

- **Baseline characteristics: Research procedure**

A total of 444 participants were randomized in a 1:1:1 ratio to three groups. Of these, 74 participants (22 in the OSS+Linaclotide group, 23 in the OSS group, and 29 in the PEG group) were subsequently excluded from the modified Intention-to-Treat (mITT) population due to failure to present for colonoscopy after randomization. The primary reasons for attrition included scheduling conflicts, resolution of symptoms leading to cancellation, withdrawal of consent, and loss to follow-up. Therefore, the final mITT population comprised 370 participants (126 in OSS+Linaclotide, 125 in OSS, and 119 in PEG). Consistent with its definition, the mITT population excludes participants who did not initiate the assigned bowel preparation regimen or undergo colonoscopy following randomization. An additional 7 participants were excluded from the PP analysis for the following reasons: 4 failed to adhere to the protocol-specified medication regimen (1 in OSS, 3 in PEG), 1 in the OSS group switched the bowel cleansing agent, and 2 in the PEG group failed to complete colonoscopy (1 due to space-occupying lesion preventing intubation, 1 due to inadequate bowel preparation). Consequently, the PP analysis included 363 participants (126 in OSS+linaclotide, 123 in OSS, and 114 in PEG) (Supplement Figure 1).

- **Supplement Table**

Supplement Table 1：Baseline characteristics (per-protocol population).

| **Characteristics** | **OSS + Linaclotide**  **（n=126）** | **OSS Group**  **（n=123）** | **PEG Group**  **（n=114）** |
| --- | --- | --- | --- |
| Age，yr | 45.0 (35.0-60.0) | 44 (34.0-61.0) | 47.5 (34.0-60.0) |
| Sex |  |  |  |
| Male | 69 (54.8) | 75 (61.0) | 63 (55.3) |
| Female | 57 (45.2) | 48 (39.0) | 51 (44.7) |
| BMI, kg/m^2^ | 23.7 (21.6-26.1) | 23.9 (21.5-25.7) | 23.7 (21.6-26.2) |
| Medical history |  |  |  |
| Drinking | 29 (23.0) | 34 (27.6) | 35 (30.7) |
| Smoking | 31 (24.6) | 30 (24.4) | 28 (24.6) |
| Hypertension | 20 (15.9) | 18 (14.6) | 19 (16.7) |
| Diabetes | 5 (4.0) | 6 (4.9) | 3 (2.6) |
| History of abdominal surgery | 26 (20.6) | 24 (19.5) | 23 (20.2) |
| History of CRC resection | 22 (17.5) | 19 (15.4) | 15 (13.2) |
| Constipation | 11 (8.7) | 9 (7.3) | 11 (9.6) |
| Others | 17 (13.5) | 18 (14.6) | 22 (19.3) |
| First colonoscopy examination |  |  |  |
| Yes | 82 (65.1) | 72 (58.5) | 70 (61.4) |
| No | 44 (34.9) | 51 (41.5) | 44 (38.6) |
| Indication of colonoscopy |  |  |  |
| Screening | 58(46.0) | 51(41.5) | 52 (45.6) |
| Diagnosis | 43 (34.1) | 44 (35.8) | 44 (38.6) |
| Surveillance | 25 (19.8) | 28 (22.8) | 18 (15.8) |

OSS+Linaclotide Group: Oral sulfate solution + 290ug Linaclotide (guanylate cyclase-C agonist, promoting intestinal secretion); OSS: Oral sulfate solution alone; PEG: Polyethylene glycol; BMI: Body mass index.

*Screening: Screening or Physical Examination. This mainly includes individuals requesting colonoscopy due to reasons such as elevated tumor markers, esophagitis, gastritis, gastric ulcer, and other gastric symptoms. Diagnosis: Diagnosis with Definite Symptoms. This includes abdominal symptoms such as diarrhea, abdominal pain, occult blood or blood in stool, weight loss, etc. Surveillance: Follow-up or Re-examination. This applies to individuals with a history of enteritis, intestinal polyps, tumors, surgery, or hemorrhoids.

Data are presented as [Median (IQR)] or n(%).

Supplement Table 2: Three groups Boston Bowel Preparation Scale score.

| **Variables** | **OSS+Linaclotide** | **OSS Group** | **PEG Group** | **Total p** | **p^*^** | **p^&^** | **p^#^** |
| --- | --- | --- | --- | --- | --- | --- | --- |
| Modified intention-to-treat analysis | n=126 | n=125 | n=119 |  |  |  |  |
| BBPS score,median (IQR) |  |  |  |  |  |  |  |
| Total | 7 (6-8) | 7 (6-8) | 6 (6-7) | ＜0.001 | 0.001 | 1.000 | ＜0.001 |
| Right colon | 2 (2-3) | 2 (2,3) | 2 (2-2) | ＜0.001 | 0.009 | 1.000 | ＜0.001 |
| Mid colon | 2 (2-3) | 2 (2-3) | 2 (2-2) | ＜0.001 | 0.004 | 0.708 | ＜0.001 |
| Left colon | 2 (2-3) | 2 (2-3) | 2 (2-2) | 0.010 | 0.034 | 1.000 | 0.019 |
| Per-protocol analysis | n=126 | n=123 | n=114 |  |  |  |  |
| BBPS score,median (IQR) |  |  |  |  |  |  |  |
| Total | 7 (6-8) | 7 (6-8) | 6 (6-7) | ＜0.001 | 0.001 | 0.411 | ＜0.001 |
| Right colon | 2 (2-3) | 2 (2-3) | 2 (2-2) | 0.001 | 0.006 | 0.328 | ＜0.001 |
| Mid colon | 2 (2-3) | 2 (2-3) | 2 (2-2) | ＜0.001 | 0.004 | 0.242 | ＜0.001 |
| Left colon | 2 (2-3) | 2 (2-3) | 2 (2-2.25) | 0.032 | 0.030 | 0.939 | 0.016 |

OSS+Linaclotide Group: Oral sulfate solution + 290ug Linaclotide (guanylate cyclase-C agonist, promoting intestinal secretion); OSS: Oral sulfate solution alone; PEG: Polyethylene glycol; BBPS: Boston Bowel Preparation Scale; Adequate bowel preparation rate was defined by BBPS score of ≥ 6, segmental BBPS ≥ 2.

*p*^*^：OSS+Linaclotide Group vs PEG Group; *p*^&^：OSS+Linaclotide Group vs OSS Group; *p*^#^：OSS Group vs PEG Group.

Data are presented as [Median (IQR)].

Supplement Table 3: Tolerance assessment (modified intention-to-treat population).

| **Variables** | **OSS+Linaclotide （n=126）** | **OSS Group**  **（n=125）** | **PEG Group**  **（n=119）** | **p-value** |
| --- | --- | --- | --- | --- |
| Taste Satisfaction,n(%) |  |  |  | 0.476 |
| Best | 91 (72.2) | 89 (71.2) | 78 (65.5) |  |
| Worse | 35 (27.8) | 36 (28.8) | 41 (34.5) |  |
| Educational methods satisfaction | 125 (99.2) | 122 (97.6) | 116 (97.5) | 0.536 |
| Sleep quality, n (%) |  |  |  | 0.323 |
| Best | 76 (60.3) | 67 (53.6) | 71 (59.7) |  |
| Same as before | 19 (15.1) | 32 (25.6) | 24 (20.2) |  |
| Worse | 31 (24.6) | 26 (20.8) | 24 (20.2) |  |
| Willing to repeat,n(%) | 97 (77.0) | 99 (79.2) | 86 (72.3) | 0.432 |
| Compliance, n (%) | 126 (100.0） | 123 (98.4) | 116 (97.5） | 0.223 |
| Cecal intubation rate, n (%) | 126 (100.0) | 125 (100.0) | 117 (98.3) | 0.120 |

OSS+Linaclotide Group: Oral sulfate solution + 290ug Linaclotide (guanylate cyclase-C agonist, promoting intestinal secretion); OSS: Oral sulfate solution alone; PEG: Polyethylene glycol.

Data are presented as n(%).

Supplement Table 4: Colonoscopy findings (per-protocol population).

| **Variables** | **OSS+Linaclotide**  **（n=126）** | **OSS Group**  **（n=123）** | **PEG Group**  **（n=114）** | **p-value** |
| --- | --- | --- | --- | --- |
| Polyp detection rate, n (%) | 55 (43.7) | 56 (45.5) | 48 (42.1) | 0.868 |
| Adenoma detection rate, n (%) | 23 (18.3) | 26 (21.1) | 18 (15.8) | 0.568 |
| Cancer detection rate, n (%) | 1 (0.8) | 1 (0.8) | 2 (1.8) | 0.740 |

OSS+Linaclotide Group: Oral sulfate solution + 290ug Linaclotide (guanylate cyclase-C agonist, promoting intestinal secretion); OSS: Oral sulfate solution alone; PEG: Polyethylene glycol.

Data are presented as n(%).

Supplement Table 5：Adverse events (modified intention-to-treat population).

| **Variables** | **OSS+Linaclotide （n=126）** | **OSS Group**  **（n=125）** | **PEG Group**  **（n=119）** | **p-value** |
| --- | --- | --- | --- | --- |
| Adverse events, n (%) |  |  |  |  |
| Total | 45 (35.7) | 59 (47.2) | 60 (50.4) | 0.050 |
| Nausea | 28 (22.2) | 37 (29.6) | 42 (35.3) | 0.077 |
| Vomiting | 12 (9.5) | 12 (9.6) | 16 (13.4) | 0.532 |
| Bloating | 5 (4.0) | 20 (16.0) | 14 (11.8) | 0.007 |
| Abdominal pain | 4 (3.2) | 8 (6.4) | 6 (5.0) | 0.491 |

OSS+Linaclotide Group: Oral sulfate solution + 290ug Linaclotide (guanylate cyclase-C agonist, promoting intestinal secretion); OSS: Oral sulfate solution alone; PEG: Polyethylene glycol.

Data are presented as n(%).

Supplement Table 6: Three groups bowel bubble score (per-protocol population).

| **Variables** | **OSS+Linaclotide**  **（n=126）** | **OSS Group**  **（n=123）** | **PEG Group**  **（n=114）** | **p-value** |
| --- | --- | --- | --- | --- |
| Left colon | 1.19±0.43 | 1.15±0.42 | 1.20±0.46 | 0.447 |
| Transverse colon | 1.19±0.43 | 1.13±0.38 | 1.22±0.49 | 0.263 |
| Right colon | 1.21±0.44 | 1.13±0.38 | 1.24±0.50 | 0.139 |

OSS + Linaclotide Group: Oral sulfate solution + 290ug Linaclotide (guanylate cyclase-C agonist, promoting intestinal secretion); OSS: Oral sulfate solution alone; PEG: Polyethylene glycol.

Data are presented as mean ± SD.

Supplement Table 7: Sensitivity analyses of adequate bowel preparation rate.

| **Variables** | **OSS+Linaclotide** | **OSS Group** | **PEG Group** | **Total p** | | **p^*^** | **p^&^** | **p^#^** |
| --- | --- | --- | --- | --- | --- | --- | --- | --- |
| Sensitivity analyses |  |  |  |  |  | |  |  |
| Primary Analysis (mITT) | 109/126 (86.5%) | 110/125 (88.0%) | 88/119 (73.9%) | 0.006 | 0.013 | | 0.723 | 0.005 |
| Best-case Analysis | 131/148 (88.5%) | 133/148 (89.9%) | 117/148 (79.1%) | 0.015 | 0.027 | | 0.708 | 0.010 |
| Worst-case Analysis | 109/148 (73.6%) | 110/148 (74.3%) | 88/148 (59.5%) | 0.008 | 0.010 | | 0.895 | 0.007 |

OSS + Linaclotide Group: Oral sulfate solution + 290ug Linaclotide (guanylate cyclase-C agonist, promoting intestinal secretion); OSS: Oral sulfate solution alone; PEG: Polyethylene glycol.

*p*^*^：OSS+Linaclotide Group vs PEG Group; *p*^&^：OSS+Linaclotide Group vs OSS Group; *p*^#^：OSS Group vs PEG Group.

Best-case analysis: all 74 participants were assumed to have adequate bowel preparation. Worst-case analysis: all 74 participants were assumed to have inadequate bowel preparation.


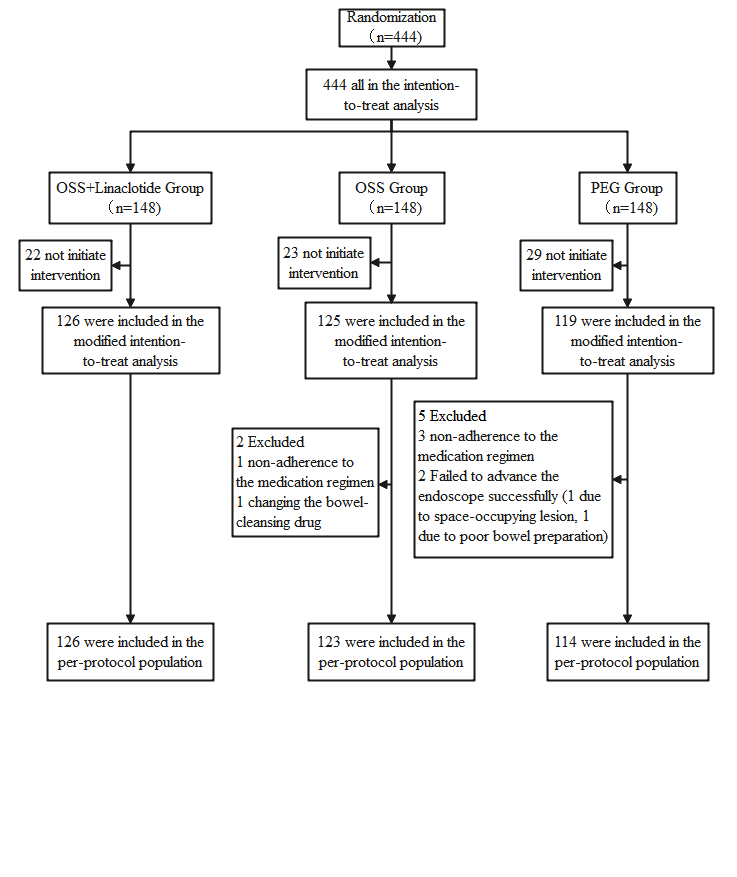


Supplement Figure 1：Flowchart of the study


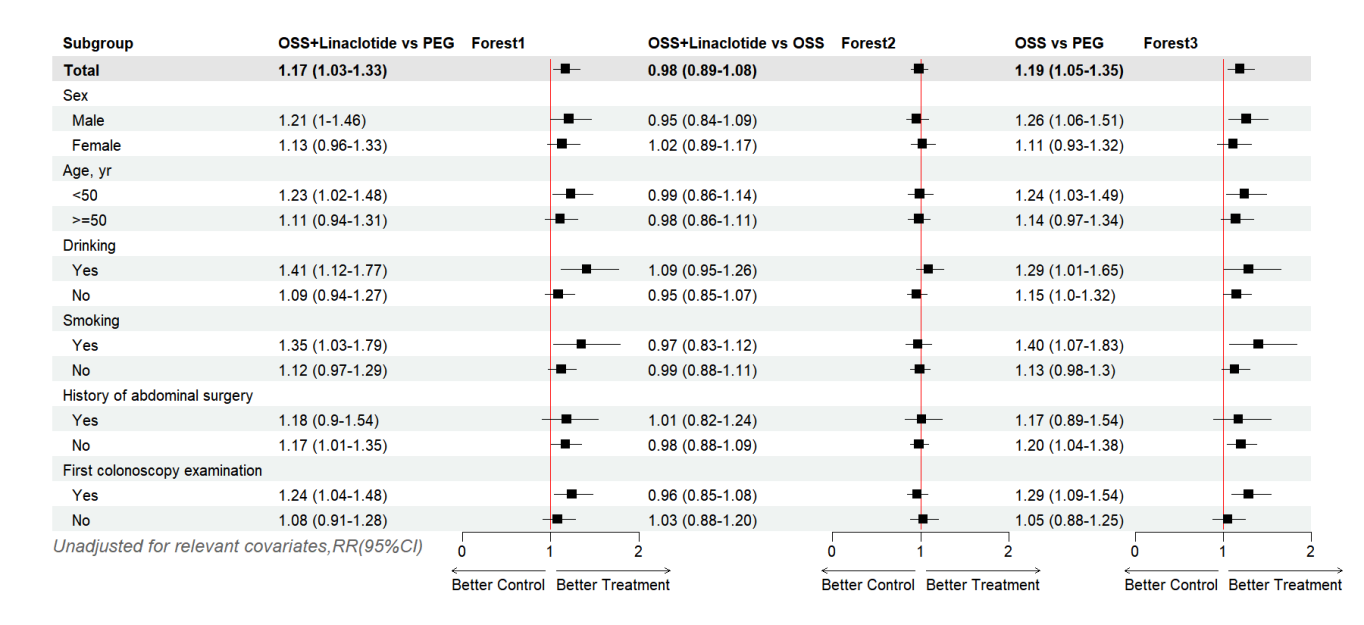
Supplement Figure 2：Subgroup analysis of bowel preparation quality (modified intent-to-treat population).

The forest plot shows the unadjusted risk ratio(RR) and 95%CI between the three intervention groups for bowel preparation quality.
