# Supplementary material for: Application of oral sulfate solution combined with linaclotide in bowel preparation for colonoscopy
Source: Front Med (Lausanne). 2026 Mar 10;13:1696298. doi: 10.3389/fmed.2026.1696298 (PMC13014616; doi:10.3389/fmed.2026.1696298)
Supplement: Supplementary file 2 [file Supplementary_file_2.docx]

# Application of Oral Sulfate Solution Combined with Linaclotide in Bowel Preparation for Colonoscopy

**TRIAL PROTOCOL**

**Principal Investigators**

Ji Xuan,MD

1 Jinling Clinical Medical College，Nanjing Medical University，Nanjing,China

2 Jinling Hospital, Affiliated Hospital of Medical School, Nanjing University，Nanjing,China

Email：xuanji @nju.edu.cn

**Protocol Version: 1.0**

**Date 2023 June 16**

**Contents**

[**1. List of Abbreviations and acronyms 1**](#_Toc10572)

[**2. Investigators 3**](#_Toc16251)

[**3. Study Synopsis 5**](#_Toc27806)

[**4. Flow Chart 11**](#_Toc2001)

[**5. Schedule of Outcome Assessments 12**](#_Toc14671)

[**6. Background 13**](#_Toc6852)

[**6.1 Research Status of Bowel Preparation for Colonoscopy 13**](#_Toc22283)

[**6.2 OSS in Bowel Preparation 14**](#_Toc30963)

[**6.3 OSS + Linaclotide in Bowel Preparation 15**](#_Toc25843)

[**7. Research Hypothesis 15**](#_Toc27126)

[**7.1 Primary Hypothesis 15**](#_Toc1365)

[**7.2 Secondary Hypotheses 15**](#_Toc25521)

[**8. Objectives 16**](#_Toc26662)

[**9. Study Design 16**](#_Toc10588)

[**10. Patient Population 16**](#_Toc12912)

[**10.1 Inclusion Criteria 16**](#_Toc18370)

[**10.2 Exclusion Criteria 16**](#_Toc29200)

[**11. Drugs 17**](#_Toc133)

[**12. Intervention 17**](#_Toc2165)

[**13. Sample Size 19**](#_Toc1127)

[**14. Randomization 19**](#_Toc3134)

[**15. Blinding and Masking 19**](#_Toc7394)

[**16. Outcomes 19**](#_Toc24811)

[**16.1 Primary Outcome 19**](#_Toc10145)

[**16.2 Secondary Outcomes 20**](#_Toc32000)

[**17. Assessment of Outcomes 20**](#_Toc6801)

[**17.1 Primary Outcome Assessment 20**](#_Toc23110)

[**17.1.1 Boston Bowel Preparation Scale(BBPS) 20**](#_Toc15777)

[**17.2 Secondary Outcomes Assessment 21**](#_Toc10511)

[**17.2.1 Bowel Bubble Score(BBS) 21**](#_Toc28531)

[**17.2.2 Tolerability 21**](#_Toc26285)

[**17.2.3 Colonoscopy findings 22**](#_Toc13440)

[**17.3 Assessment of Safety 22**](#_Toc14451)

[**17.4 Adverse Event Definitions 22**](#_Toc3204)

[**17.4.1 Adverse Event 22**](#_Toc14336)

[**17.4.2 Serious Adverse Event 23**](#_Toc10288)

[**17.5 Clinical Management of Adverse Events 24**](#_Toc676)

[**17.5.1 Identification of Adverse Events by the Investigator 24**](#_Toc10604)

[**17.5.2 Reporting of Adverse Events 24**](#_Toc11546)

[**17.5.3 Prompt Reporting of Serious Adverse Events 25**](#_Toc10931)

[**18. Study Discontinuation Criteria 25**](#_Toc1520)

[**19. Study Exit Criteria 25**](#_Toc28241)

[**20. Study Exclusion Criteria 26**](#_Toc9299)

[**21. Statistical Analysis 26**](#_Toc23690)

[**21.1 Analysis Populations 26**](#_Toc28188)

[**21.1.1 Intention-to-treat Population 26**](#_Toc28656)

[**21.1.2 Modified Intention-to-Treat (mITT) Population 27**](#_Toc22345)

[**21.1.3 Safety Population 27**](#_Toc26993)

[**21.2 Analysis of Primary Efficacy Outcome 27**](#_Toc28238)

[**21.3 Analysis of Secondary Efficacy Outcomes 27**](#_Toc12780)

[**21.4 Analysis of Safety Outcomes 28**](#_Toc9185)

[**21.5 Handling of Missing Data 28**](#_Toc15689)

[**21.6 Covariates and Planned Subgroups 29**](#_Toc29227)

[**22. Data Safety Monitoring Board 30**](#_Toc9061)

[**23. Ethical and Regulatory Consideration 30**](#_Toc26248)

[**23.1 General Requirements and Considerations 30**](#_Toc1366)

[**23.2 Study Monitoring and Quality Control 30**](#_Toc27196)

[**23.3 Informed Consent 30**](#_Toc18893)

[**23.4 Confidentiality 31**](#_Toc18879)

[**24. Administrative Procedures 31**](#_Toc20906)

[**24.1 Secrecy Agreement 31**](#_Toc14230)

[**24.2 Ownership of Data and Use of the Study Results 31**](#_Toc3575)

[**24.3 Protocol Amendments 32**](#_Toc11990)

[**25. Data Retention 32**](#_Toc27361)

[**26. Study Report 32**](#_Toc31418)

[**27. Publications 32**](#_Toc24433)

[**28. References 34**](#_Toc5111)

1. **List of Abbreviations and acronyms**

| **Abbreviations /acronyms** | **Definitions** |
| --- | --- |
| ADR | Adenoma Detection Rate |
| AE | Adverse Event |
| BBPS | Boston Bowel Preparation Scale |
| BBS | Bowel Bubble Score |
| BMI | Body Mass Index |
| BP | Blood Pressure |
| CRF | Case Report Form |
| CI | Confidence Interval |
| DSMB | Data Safety Monitoring Board |
| ESGE | European Society of Gastrointestinal Endoscopy |
| GEE | Generalized Estimating Equation |
| GCP | Good Clinical Practice |
| ICH-E9 | International Conference on Harmonization - Statistical principles for Clinical Trials |
| ITT | Intention-to-Treat |
| IPTW | Inverse-Probability Treatment Weighting |
| mITT | Modified Intention-to-Treat |
| OSS | Oral Sulfate Solution |
| PDR | Polyp Detection Rate |
| PEG | Polyethylene Glycol |
| PP | Per-protocol |
| RCT | Randomized Controlled Trial |
| RR | Risk Ratio |
| SAP | Statistical Analysis Plan |
| SAE | Serious Adverse Event |
| TEAEs | Treatment-Emergent Adverse Events |

1. **Investigators**

| **Ji Xuan**  Chief Physician, Department of Gastroenterology  1 Jinling Clinical Medical College, Nanjing Medical University  2 Jinling Hospital, Affiliated Hospital of Medical School, Nanjing University | **Yuxiu Liu**  Data and Statistics Division, Research Fellow  Jinling Hospital, Medical School of Nanjing University |
| --- | --- |
| **Mei Shao**  Department of Gastroenterology, Doctor  Jinling Hospital, Affiliated Hospital of Medical School, Nanjing University | **Haifeng Lan**  Department of Gastroenterology, Master's student  Department of Gastroenterology, Jinling Clinical Medical College, Nanjing Medical University |
| **Mengjie Lu**  Health Science Center, Research Fellow  Ningbo University | **Shupei Li**  Department of Gastroenterology, Master's student in Pharmaceutical Sciences  Jinling School of Clinical Medicine,Nanjing University of Chinese Medicine |
| **Ya Yang**  Department of Gastroenterology, Doctor  Jinling Hospital, Affiliated Hospital of Medical School, Nanjing University | **Qi Zhai**  Department of Gastroenterology, Doctor  Jinling Hospital, Affiliated Hospital of Medical School, Nanjing University |
| **Qing Gao**  Department of Gastroenterology, Nurse  Jinling Hospital, Affiliated Hospital of Medical School, Nanjing University |  |

1. **Study Synopsis**

| **Study Title** | Application of Oral Sulfate Solution Combined with Linaclotide in Bowel Preparation for Colonoscopy |
| --- | --- |
| **Indication** | Bowel preparation |
| **Study registry number** | Clinical trials : NCT06091735 |
| **Number of Subjects** | 444(148 subjects per group) |
| **Study Estimate Duration** | June 2023 until June 2024 |
| **Research Unit** | Department of Gastroenterology, Jinling Hospital, Affiliated Hospital of Medical School, Nanjing University. |
| **Objectives** | This study aims to explore the application effect of Oral sulfate solution (OSS) combined with linaclotide in bowel preparation. |
| **Study Design** | Single-center, single-blind, randomized controlled clinical study |
| **Study Population** | 18-80 years, colonoscopy patients |
| **Inclusion Criteria** | - Age 18-80 years，either sex; - Individuals scheduled to undergo elective colonoscopy; - Capable of adhering to the study protocol, follow-up visits, and completion of all study-related assessments (including validated scales). |
| **Exclusion Criteria** | - Severe cardiopulmonary, hepatic, or renal dysfunction, including:Severe heart failure (New York Heart Association Class III or IV), recent acute myocardial infarction, or unstable angina;Uncontrolled hypertension (systolic BP ≥160 mmHg or diastolic BP ≥100 mmHg despite antihypertensive therapy); Chronic liver disease, chronic kidney disease, cirrhosis, or ascites; - Suspected gastrointestinal obstruction or perforation; - Pregnant or lactating women, or individuals planning pregnancy during the study period; - Mental illness or physical disability; - Allergic to bowel preparation drugs; - Failure to undergo colonoscopy after bowel preparation completion; - Inability to tolerate diagnostic colonoscopy; - Voluntary withdrawal; - Participation in another clinical trial within 3 months prior to enrollment or during the study period. |
| **Drugs** | OSS+Linaclotide group：Oral Sulfate Solution (OSS, 177 mL/bottle; packaged as 2 bottles per box)+ 290ug linaclotide.  OSS group：Oral sulfate solution alone, uncombined linaclotide.  PEG group：2 bags of compound polyethylene glycol. |
| **Randomization** | Eligible patients will be consecutively  randomized to treatment with OSS+Linaclotide group, OSS group, PEG group with a  ratio of 1:1:1 by a web-based APP (Jinlingshu) on  mobile phone or computer  (https://jinlingshu.com/). Randomization will be  stratified by participating centres permutation  block size of 6. |
| **Intervention** | All participants received face-to-face instruction from the research team, covering medication administration methods, dosages, timing, management of adverse reactions, and potential consequences of inadequate bowel preparation. Reference videos were provided to aid comprehension. The detailed medication regimen was as follows: On the evening before colonoscopy, participants consumed dinner before 18:00. At 20:00, they began bowel cleansing agent dissolved in water, followed by approximately 500 mL of water consumed evenly over the next 2 hours (total fluid intake: 1.5 L). Six hours prior to the scheduled colonoscopy time, participants ingested the second dose of the bowel cleansing agent and one 20 mL bottle of simethicone emulsion, followed by 1.5 L of consumed within 2 hours. Immediately before colonoscopy, an additional bottle of simethicone was administered. For the OSS+Linaclotide group, participants first orally took one 290ug linaclotide capsule at 18:00 on the evening prior to colonoscopy, with subsequent steps identical to the other groups. All groups adhered to the "6+2" protocol: strict fasting for 6 hours and strict fluid restriction for 2 hours prior to colonoscopy. |
| **Consent** | Written informed consent must be obtained from participants or their legally authorized representatives before any study procedures. |
| **Primary and secondary outcome measures** | - Primary outcome:   The primary outcome was the efficacy of bowel cleansing, assessed using the Boston Bowel Preparation Scale (BBPS). Total BBPS scores and segmental scores (right, transverse, and left colon) were compared across the three groups. Successful bowel preparation was defined as: A total BBPS score ≥6, and a score ≥2 for each individual segment.The adequate rate was calculated as: Successful rate=Successful prepared cases/Total cases ×100%.   - Secondary outcomes:  1. Bowel bubble Score; 2. Tolerability  - Taste satisfaction; - Promote education satisfaction; - Sleep quality; - Willingness to repeat; - Compliance;  1. Colonoscopy findings  - Polyp detection rate; - Adenoma detection rate; - Cancer detection rate. |
| **Safety outcome measures** | The proportion of patients experiencing nausea, vomiting, abdominal distension and abdominal pain or other serious adverse events. |
| **Sample Size Calculation** | Based on literature data from similar study designs, we set the expected adequate bowel preparation rates for the OSS+Linaclotide group, OSS monotherapy group, and PEG group at 90%, 86%, and 69.36%, respectively. A two-sided significance level of α=0.05 and a statistical power of 80% were established, with samples allocated in a 1:1:1 ratio across the three groups. The minimum required sample size per group was calculated as 118 participants. Accounting for a 20% dropout rate, the planned sample size per group was at least 148 participants. These calculations were performed using PASS software (version 15.0; NCSS, LLC, Kaysville, UT, USA). |
| **Statistical Methods** | All efficacy analyses will be conducted on data  from all randomly assigned patients according to  the intention-to-treat (ITT) principle. All efforts will be made to minimize the amount of missing data. Analyses will also be repeated according the per protocol principle. For the primary outcome analysis, the proportions of adequate bowel preparation will be compared 3 group arms using modified Poisson regression model. Both adjusted and unadjusted risk ratio and their 95% CIs will be reported. In case of non-convergence of the covariate-adjusted model, the inverse-probability treatment weighting (IPTW) method will be used. We will analyze the primary outcome in the modified ITT population, and repeat the analysis in the PP population. For shedding cases, follow-up will be performed until the end of the study, and the results will be included in the final analysis. Statistical analysis will be performed on the SAS 9.4 system and R version 4.5.0. Details of these are provided in the Statistical Analysis Plan.  Secondary outcomes, adverse events (AE) and serious adverse event (SAE) will be summarized and presented by treatment group for all patients. Continuous variables will be presented with mean and SD, and compared using t- tests. Categorical variables will be described in frequency and percentage, and compared using the χ2 or Fisher’s exact test. |

1. **Flow Chart**

**Population**

- Age 18-80 years;
- Individuals scheduled to undergo elective colonoscopy.

**Inclusion and**

**exclusion criteria**

**Randomization**

**1:1:1**

**Bowel Preparation**

**OSS+Linaclotide**

**PEG group**

- **Primary Outcome**

Adequacy rate of bowel preparation.

- **Secondary Outcomes**

Bowel bubble Score;

Tolerability

Colonoscopy findings

**OSS group**

Figure 1：Research flowchart

1. **Schedule of Outcome Assessments**

| **Time points Assessments** | **Baseline** | | **Completion of bowel preparation** | | |
| --- | --- | --- | --- | --- | --- |
|  | **Recruiting** | **Randomisation** | **On the Day of Colonoscopy** | **During colonoscopy** | **Within 30 days after polypectomy** |
| **Baseline data** |  |  |  |  |  |
| Informed consent | 🗸 |  |  |  |  |
| Inclusion and exclusion criteria |  | 🗸 |  |  |  |
| Population information |  |  | 🗸 |  |  |
| Medical history and treatment history |  |  | 🗸 |  |  |
| **Primary Outcome** |  |  |  |  |  |
| Bowel cleansing efficacy |  |  |  | 🗸 |  |
| **Secondary Outcomes** |  |  |  |  |  |
| Bowel Bubble Score |  |  |  | 🗸 |  |
| Tolerability |  |  | 🗸 |  |  |
| Satisfaction |  |  | 🗸 |  |  |
| Sleep quality |  |  | 🗸 |  |  |
| Willing to repeat |  |  | 🗸 |  |  |
| Compliance |  |  | 🗸 |  |  |
| Cecal intubation rate |  |  |  | 🗸 |  |
| Colonoscopy findings(ADR, PDR) |  |  |  | 🗸 |  |
| **Safety Outcomes** |  |  |  |  |  |
| Nausea |  |  | 🗸 |  |  |
| Vomiting |  |  | 🗸 |  |  |
| Bloating |  |  | 🗸 |  |  |
| Abdominal pain |  |  | 🗸 |  |  |
| Others |  |  | 🗸 |  |  |
| Complications |  |  |  |  | 🗸 |

1. **Background**

Electronic colonoscopy is the most important method for diagnosing colorectal diseases, and the quality of its examination is closely related to the effect of bowel preparation. High-quality bowel preparation can increase the detection rate of colonoscopy lesions and the safety of endoscopic treatment^[1,2]^. The selection and correct use of bowel cleansers are one of the important influencing factors of the quality of bowel preparation before colonoscopy^[3]^. The ideal bowel preparation method for colonoscopy should have the following characteristics: the ability to empty the feces in the colon within a short period of time; It does not cause changes in the colonic mucosa; It will not cause discomfort to the patient. It does not cause disorders of water and electrolytes; The price is moderate^[4,5]^. At present, the commonly used bowel cleansers in clinical practice each have their own characteristics and cannot fully meet the above standards. It is necessary to select the appropriate bowel cleanser based on the specific population. Bowel preparation remains a clinical issue faced by both doctors and patients and is a hot topic in medical research.

- 1. **Research Status of Bowel Preparation for Colonoscopy**

At present, the commonly used bowel cleansers for bowel preparation mainly fall into two categories: stimulant laxatives and solubilizing laxatives. Commonly used drugs include compound polyethylene glycol (PEG), sulfate, mannitol, sodium phosphate salt, etc., as well as some other effective measures that can improve bowel preparation, such as senna leaves, castor oil, lactulose, linalopin, moapride, etc.The dosage and administration method of each laxative vary depending on the type of drug, mechanism of action, and the target population. PEG is currently the most widely used laxative both at home and abroad^[6,7]^. Foreign studies have shown that high-dose (4L) PEG has few complications and high safety, and can even be used for patients with liver and kidney function impairment and congestive heart failure. It has been recommended by the European Society of Gastrointestinal Endoscopy (ESGE) ^[2,8]^. However, large doses of intestinal cleansers do not seem to be suitable for the Chinese population. In the RCTS of Peng Cheng^[9]^ et al., the incidence of adverse events such as nausea, vomiting and abdominal distension in the 3L-PEG group was lower, and the satisfaction rate was higher than that in the 4L-PEG group, P=0.009. Studies have also shown that the effect of 3L-PEG is superior to that of 2L^[10]^. Therefore, in combination with the relevant bowel preparation guidelines for digestive endoscopy diagnosis and treatment in China^[11]^, it is currently believed that 3L-PEG is more suitable for the Chinese population to use.However, PEG laxatives have a poor taste and require a large amount of water intake, which makes patients intolerant and less compliant. 5% to 15% of patients cannot complete bowel preparation well, resulting in poor bowel preparation quality during colonoscopy and affecting the observation effect.

- 1. **OSS in Bowel Preparation**

Oral sulfate solution (OSS) is a new type of bowel cleaner in China. Its active ingredient is sulfate, which, like PEG, is an osmotic laxative and is not digested or absorbed by the intestines. It relies on sulfate ions to provide osmotic pressure. Different from traditional magnesium sulfate preparations, when cleaning the intestines, it simultaneously supplements sodium ions and potassium ions, reducing the risk of water and electrolyte imbalance. It utilizes the concentration of electrolytes in the bowel tract to accumulate water, causing watery diarrhea and achieving the effect of cleaning the intestinal tract^[12]^. Moreover, the improved OSS is a colorless and clear liquid with an orange flavor, which can to a certain extent enhance patients' satisfaction with the taste. Previous studies have conducted different RCT comparisons of active ingredient sulfate solutions (with different dosages and component proportions used in each study), and the conclusions drawn are still not uniform. In the Phase III clinical trial comparing OSS and 3L-PEG with the same dose and composition as in this study, scholars believed that in terms of the quality of intestinal preparation in Chinese adults, the dose-fractionated OSS regimen was not inferior to the dose-fractionated 3-L-PEG regimen, and the safety and acceptability of the two groups were similar^[13]^. OSS and 2L-PEG/Asc (ascorbic acid) are equally effective in successful intestinal cleansing and have acceptable tolerance^[14]^. In conclusion, OSS is a promising and relatively safe alternative to colonoscopy^[15,16]^.The new type of OSS is a newly launched improved drug in China with a good taste. So far, there have been no randomized controlled clinical studies mainly focusing on the oral concentrated solution of the new type of oral sulfate solution.

- 1. **OSS + Linaclotide in Bowel Preparation**

Linaclotide is a guanylate cyclase C receptor agonist, which can promote bowel fluid secretion, increase the water content of feces, facilitate the accumulation of fluid in the intestinal lumen, and promote the opening of chloride ion channels, enhance bowel motility, and increase the frequency of defecation. Previous studies have confirmed its safety and efficacy in bowel preparation by combining it with other bowel cleansing drugs^[17,18]^. Research shows^[19]^ that linaclotide combined with PEG can improve the quality of bowel preparation in the colon, shorten the time of first defecation, and enhance patient satisfaction and comfort.Inspired by this, it is proposed whether the new OSS can be combined with linaclotide for intestinal cleaning.After literature search, there have been no reports on the combined use of OSS and linaclotide for bowel cleaning. Therefore, in order to further optimize the bowel preparation plan for colonoscopy. This study is the first to combine OSS with linaclotide for intestinal cleansing, filling the scientific research gap in this direction.

In conclusion, the choice of bowel cleansers remains a pain point for both patients and clinicians. Therefore, this study intends to conduct a single-center, single-blind, randomized controlled clinical study of OSS combined with linaclotide. At the same time, it will observe the number of steps taken during exercise and fatigue curves to explore the efficacy and safety of OSS combined with linaclotide. This method is expected to become another new personalized bowel cleansing plan for bowel preparation.

1. **Research Hypothesis**
   1. **Primary Hypothesis**

The cleansing efficacy of 290 µg linaclotide combined with OSS is non- inferior to that of OSS，superior to the traditional PEG group.

- 1. **Secondary Hypotheses**

The tolerance and safety of the OSS+Linaclotide group were superior to those of the OSS group and the PEG group.

1. **Objectives**

This study aimed to evaluate the efficacy and safety of OSS combined with linaclotide as a bowel preparation regimen prior to colonoscopy. The findings may have immediate clinical implications. If the results are positive, this regimen could provide a novel, effective, and safe bowel cleansing option, while offering evidence for the application of linaclotide in bowel preparation.

1. **Study Design**

This study is a investigator-initiated, single-center, single-blind, randomized controlled trial. This study strictly adheres to the principles of the Declaration of Helsinki and has been approved by the Medical Ethics Committee of our hospital (Ethics Number: DZQQ-KYLL-23-11), and has been registered on ClinicalTrials.gov (Registration Number: NCT06091735).

1. **Patient Population**

All potential participants will undergo standardized clinical evaluation by qualified site investigators to verify eligibility. Following comprehensive review of medical histories and baseline assessments, subjects meeting all predefined inclusion/exclusion criteria will be formally enrolled after obtaining documented informed consent. Trained study physicians will conduct the consent process, providing complete disclosure of study objectives, experimental procedures, potential risks/benefits, and alternative therapeutic options. Written informed consent will be obtained only after confirming participants' full understanding of the study protocol and their rights as research subjects.

- 1. **Inclusion Criteria**
- Age 18-80 years，either sex;
- Individuals scheduled to undergo elective colonoscopy;
- Capable of adhering to the study protocol, follow-up visits, and completion of all study-related assessments (including validated scales).
  1. **Exclusion Criteria**
- Severe cardiopulmonary, hepatic, or renal dysfunction, including:Severe heart failure (New York Heart Association Class III or IV), recent acute myocardial infarction, or unstable angina;Uncontrolled hypertension (systolic BP ≥160 mmHg or diastolic BP ≥100 mmHg despite antihypertensive therapy); Chronic liver disease, chronic kidney disease, cirrhosis, or ascites;
- Suspected gastrointestinal obstruction or perforation.
- Pregnant or lactating women, or individuals planning pregnancy during the study period;
- Mental illness or physical disability;
- Allergic to bowel preparation drugs;
- Failure to undergo colonoscopy after bowel preparation completion;
- Inability to tolerate diagnostic colonoscopy;
- Voluntary withdrawal;
- Participation in another clinical trial within 3 months prior to enrollment or during the study period.

1. **Drugs**

The Oral Sulfate Solution (OSS), manufactured by Jichuan Pharmaceutical Group Co., Ltd. (specification: 177 mL/bottle; packaged as 2 bottles per box), contains 17.5 g sodium sulfate, 3.13 g potassium sulfate, and 1.6 g magnesium sulfate per 177 mL bottle. Linaclotide (produced by Almac; available as 290-microgram capsules) is also used. Polyethylene glycol (PEG), supplied by Shenzhen Wanhe Pharmaceutical Co., Ltd. (specification: 68.56 g/bag), consists of 1.46 g sodium chloride, 5.68 g anhydrous sodium sulfate, 0.74 g potassium chloride, 1.68 g sodium bicarbonate, and 59 g polyethylene glycol 4000 (PEG-4000) per bag.

1. **Intervention**

Participants were provided with both paper-based instructions and videos detailing the bowel preparation protocols. Covering topics such as the purpose of bowel preparation, dietary restrictions, administration of bowel preparation agents and potential discomfort after medication intake, along with corresponding treatment options. All participants are instructed to adhere to a low- fibre diet on preoperative 1-3 days^[20]^.

The specific medication guidelines are outlined as follows: Dinner should be completed before 18:00 on the evening prior to the examination. At 20:00, commence the administration of the laxative by dissolving one bottle (or pack) of the intestinal cleanser in water and consuming it. Subsequently, consume approximately 500 mL of water every 15 to 30 minutes for a duration of 2 hours, totaling 1.5 L of water intake. Six hours prior to the colonoscopy examination, dissolve the second bottle (or pack) of the intestinal cleanser and a 20-mL bottle of dimethicone emulsion in water and ingest the mixture, followed by drinking an additional 1.5L of water within 2 hours. During the preparation stage for the examination, take another bottle of dimethicone. For participants in the OSS + Linaclotide group, administer one 290μg dose of linaclotide orally at 18:00 on the day preceding the examination. The subsequent steps for this group are identical to those outlined above (Figure 1). All groups adhere to the "6 + 2" protocol, which entails strictly fasting for 6 hours and abstaining from fluid intake for 2 hours prior to the examination.

All three groups adhered to a regimen of ingesting the bowel cleanser in slow, divided doses, with each dose needing to be consumed within a two-hour timeframe. It was advised that patients engage in suitable activities following medication intake to alleviate any discomfort arising from excessive water consumption.


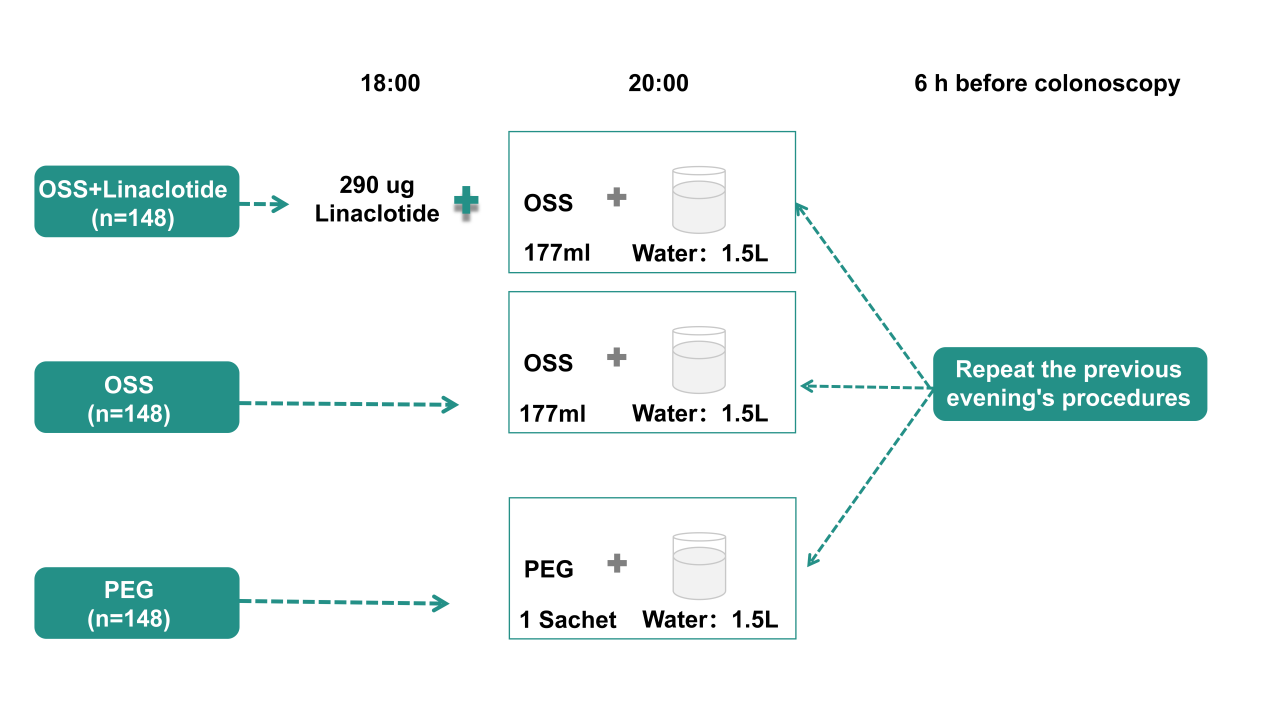


Figure 1:Bowel preparation methods

1. **Sample Size**

Drawing on data from previous research, we set the success rates for the OSS+Linaclotide group, the OSS group, and the PEG group at 90%^[21]^, 86%^[15]^, and 69.36%^[22]^, respectively. We set the significance level of the two - tailed test at α = 0.05 and the statistical power at 80%. We allocated samples to each group in a 1:1:1 ratio. The minimum sample size for each group was calculated to be 118 cases. Considering a 20% dropout rate, we plan to include at least 148 subjects in each group.This estimation was performed based on PASS (NCSS, LLC. Kaysville, Utah, USA) version 15.0.

1. **Randomization**

Randomization will be done by a web-based APP (Jinlingshu) on mobile phone or computer (https://jinlingshu.com/). The automated system will assign an appropriate set of study medication to each patient. Patients will be randomly assigned in a 1:1:1 ratio to one of three groups: OSS+Linaclotide group, OSS group, or PEG group. Randomization will be stratified by participating centre with permutation block size of 6. Randomization will be completely concealed by having both web-based real-time allocation (All drugs will have a unique number. Subjects will be assigned a random serial number according to the time they were enrolled, and corresponding masked medications will be provided).

1. **Blinding and Masking**

A single-blind (observer-blind) approach was implemented, with treatment allocations concealed from clinical assessors and data collection personnel. The DSMB reviewed unmasked safety data throughout the trial.

1. **Outcomes**
   1. **Primary Outcome**

Evaluators will assess participants at different time points. Baseline assessments include sociodemographic characteristics (age, gender and BMI) , medical history and others. The primary efficacy outcome measure was the bowel cleansing quality. One independent and experienced endoscopists (With an annual experience performing approximately 1000 endoscopic treatments, will undergo standardised training to proficiently employ the BBPS for assessing bowel preparation quality), blinded to the participant’s group allocation, will assess using the Boston Bowel Preparation Scale (BBPS), and then two researchers will review it. Total BBPS scores and segment-specific scores (right colon, transverse colon, left colon) were compared among the three groups. Adequate bowel preparation was defined as a total BBPS score ≥6 and segmental scores ≥2 in all colon segments. The adequate rate was calculated as: adequate rate=Adequately prepared cases/Total cases ×100%.

- 1. **Secondary Outcomes**
- Bowel bubble Score;
- Tolerability
- Taste satisfaction;
- Promote education satisfaction;
- Sleep quality;
- Willingness to repeat;
- Compliance;
- Colonoscopy findings
- Polyp detection rate;
- Adenoma detection rate;
- Cancer detection rate.

1. **Assessment of Outcomes**
   1. **Primary Outcome Assessment**
      1. **Boston Bowel Preparation Scale(BBPS)**

The BBPS is a standardized tool for assessing the quality of bowel preparation before colonoscopy, developed by the Boston University Medical Center^[23]^. This scale divides the colon into three parts (the right colon, the transverse colon, and the left colon), and scores each part separately, with a total score range of 0 to 9 points^[24]^.

- 0 points: The mucosa is invisible and cannot be evaluated due to solid feces or liquid covering it.
- 1 point: The mucosal part is visible, but there is residual feces/turbid fluid that affects observation (rinsing or aspiration is required).
- 2 points: A small amount of residual feces/liquid, with clearly visible mucous membranes (slight impact, no additional cleaning required).
- 3 points: The mucosa is completely visible, with no residual feces or fluid.

The primary efficacy outcome measure for this study is:Adequate rate of bowel preparation.Adequate bowel preparation was defined as a total BBPS score ≥6 and segmental scores ≥2 in all colon segments. The adequate rate was calculated as: Adequate rate=Adequately prepared cases/Total cases ×100%.We will retain the video footage and images of the patient's colonoscopy. The colonoscopy physician will first conduct a BBPS score after the colonoscopy is completed, and then two researchers will review it. Disagreements will be resolved by consensus. Electronic Data Capture System did not reveal the group assignment and patients were instructed not to reveal any relevant information that could potentially lead to disclosing their treatment group to the assessors.

The primary hypothesis to be tested is that the effect of bowel preparation after taking intestinal cleansers. The primary effectiveness analysis for this trial is The therapeutic effect differences between the OSS+Linaclotide group and the OSS group as well as the PEG group.

- 1. **Secondary Outcomes Assessment**
     1. **Bowel Bubble Score(BBS)**
- 0: No bubbles;
- 1: Mild bubble interference (<25% mucosal obscuration);
- 2: Moderate (25-50%);
- 3: Severe (>50%).

This scoring system evaluates the amount of bubbles/froth in the intestinal lumen during endoscopy. Commonly used to assess: Efficacy of pre-procedure simethicone administration and mucosal visualization quality.

- - 1. **Tolerability**

Tolerability involves patients’ subjective assessment of the bowel preparation process and willingness to repeat colonoscopy with the same bowel preparation agent. After completing bowel preparation, on the day of the colonoscopy, the investigator will give all participants the bowel preparation tolerability questionnaire. The questionnaire includes “taste(best or worse) and volume of the bowel preparation agent”, “overall experience of the bowel preparation process” and “willing to repeat”, “compliance”(Yes or No), will be recorded as a ‘yes’ or ‘no’ response (1=yes and 2=no).

- - 1. **Colonoscopy findings**

Including the detection rate of polyps, adenomas and cancers, cecal intubation rate.

- 1. **Assessment of Safety**

The safety assessments will include monitoring and recording adverse events, including serious and non-serious adverse events, measuring vital signs specified in the protocol, and other protocol specified tests that are considered to be critical to the safety evaluation of the study.

This study mainly assesses the adverse symptoms that occur during medication, including gastrointestinal symptoms such as nausea, vomiting, bloating, and abdominal pain, as well as other systemic reactions. It is recorded as “Yes” or “No”.

- 1. **Adverse Event Definitions**
     1. **Adverse Event**

Any adverse medical occurrence in a trial participant after enrollment, regardless of causal relationship to the investigational product, will be recorded as an Adverse Event (AE). This includes:

- Emergence of new symptoms or diagnoses;
- Clinically significant worsening of pre-existing conditions (in severity or frequency);
- Abnormal diagnostic test results with medical relevance;
- Pre-existing stable conditions without deterioration should not be reported as AEs.

During the study period, various adverse events were recorded and their severity was judged, with particular emphasis on observing adverse events (such as digestive tract obstruction or perforation, intestinal obstruction or gastric retention; Severe active inflammatory bowel disease or toxic megacolon; Consciousness disorder; Allergic to the drug components therein; Unable to swallow independently; After ileostomy; Chronic kidney disease, etc).

- **Criteria for Judging the Severity of Adverse Events:**
- Mild: Does not affect the normal life of the subjects;
- Moderate: To a certain extent, it affects the normal life of the subjects;
- Severe: Significantly affects the normal life of the subjects.
- **Relationship of Adverse Events:**
- Unrelated: Adverse events definitively caused by external factors (e.g., comorbidities, environmental exposures) and unrelated to drug administration per predefined criteria.
- Possibly: Clinical events (including lab abnormalities) with plausible temporal drug association but also explainable by concurrent conditions/medications; withdrawal data may be unclear.
- Probably: Clinical events temporally linked to drug use, unlikely due to other causes, with reversible response upon discontinuation.
- Related: Clinical events with reasonable evidence suggesting drug causation, supported by temporal proximity and/or withdrawal response.
  - 1. **Serious Adverse Event**

Serious adverse events(SAE): When an adverse event meets one or more of the following conditions, regardless of whether it is related to treatment or not, it should be regarded as a serious adverse event: life-threatening; Death; Resulting in hospitalization or prolonging of hospital stay; Permanent or severe disability; It leads to congenital malformations.

Upon occurrence of an SAE, immediate medical intervention shall be initiated, and an assessment shall be conducted to determine whether the event was anticipated. If the event is determined to be a Suspected and Unexpected Serious Adverse Reaction (SUSAR), it must be reported within 24 hours to the clinical trial institution, ethics committee, drug regulatory authority, and health administration department.

- 1. **Clinical Management of Adverse Events**
     1. **Identification of Adverse Events by the Investigator**

AE monitoring and reporting will continue until completion of colonoscopy. Serious Adverse Events (SAEs) will be tracked through the final study exit visit (whichever occurs first: completed visit, death, or study end) or until the subject is lost to follow-up.

When the subjects arrive at the endoscopy center of our hospital, AE identification is collected through the hospital medical records and oral medical history of the subjects or their legally authorized agents. For the follow-up after the completion of colonoscopy, the subject (or the legally authorized representative, if the subject is unable to answer the questions) will be asked about the occurrence of AE since taking the medication. The AE that was in progress at the time of the last contact will be updated to the stop date or confirmed as in progress. AE collection will continue until the end of the colonoscopy, and SAE collection will continue until the symptoms are completely improved or the last contact is made.A consistent methodology of eliciting AEs at all subject evaluation timepoints will be used.

For AE reporting, established diagnoses (when available) supersede individual signs/symptoms. When medical characterization is not possible, all observed manifestations should be documented. However, if a constellation of signs and/or symptoms cannot be medically characterized as a single diagnosis it is acceptable to report the information that is ultimately available.

- - 1. **Reporting of Adverse Events**

In the "Case Report Form", an "Adverse Event Record Form" should be set up. Researchers should record in detail any adverse events that occurred in the enrolled cases, regardless of whether they are related to the surgical treatment method. All such events must be recorded in the original data using medical terms and copied to the case report form. The record of adverse events should include: the description of the adverse event and all related symptoms, the start time, the end time, the severity, the relationship with the trial, the measures taken and the outcome.

- - 1. **Prompt Reporting of Serious Adverse Events**

When adverse events occur, regardless of whether there is a causal relationship between the event and the study, active handling should be carried out. All adverse events should be followed up until they are properly resolved or the condition stabilizes.

Investigators must initiate telephone notification to the clinical coordinator within 1 working day of SAE awareness, followed by submission of a fully completed SAE form (signed by the investigator or qualified designee) via fax within 24 hours. Corresponding CRF entries must be made concurrently. Incomplete information does not delay initial reporting; forms shall be updated as new data become available.

Initial causality determination must accompany the first report. Subsequent amendments are permitted when post-hoc data contradict original assessments, requiring re-signature and resubmission with updated timelines.

The investigator will always provide an assessment of causality at the time of the initial report as described previously. If data obtained after reporting indicates that the assessment of causality is incorrect, then the SAE form may be appropriately amended, signed and dated, and resubmitted.

Local Ethics Committee notification must follow institutional guidelines, with supplementary investigations initiated based on clinical judgment of causative factors. This may involve specialist consultation, additional testing, or post-mortem analysis (when death occurs). Subject care must never be compromised by reporting delays.

1. **Study Discontinuation Criteria**

If a subject finds certain adverse events intolerable or the researcher discovers that the risk/benefit ratio is unacceptable to the subject personally, the researcher or the secondary researcher has the right and responsibility to discontinue the treatment of the subject. The suspension is "permanent". Once a subject withdraws, they cannot be enrolled in the trial again.

1. **Study Exit Criteria**

The subject or their legal representative (such as parents or legal guardians) withdraws the informed consent form, and the subject feels that the therapeutic effect is poor or is lost to follow-up for unknown reasons. Subjects can withdraw from the trial at any time for any reason. Researchers can no longer have direct contact with subjects to obtain new information (including data clarification forms).

1. **Study Exclusion Criteria**

After inclusion, if it was found that the subjects did not meet the inclusion criteria or were mistakenly included due to meeting any of the exclusion criteria, the subjects were unable to provide follow-up data on time and accurately, and the subjects withdrew the informed consent form.

1. **Statistical Analysis**
   1. **Analysis Populations**

The modified intention-to-treat (mITT) population and Per-protocol (PP) population will be analyzed. Patients who withdraw informed consent immediately after randomization and do not receive any treatment should be excluded from all analysis populations. The following analysis populations are planned for the studies:

- - 1. **Intention-to-treat Population**

The ITT population will consist of all patients who signed the informed consent and are randomized in the study, and did not withdraw informed consent immediately after randomization and before any study treatment. The data from the ITT population will be analyzed by the treatment group assignment given at the time of randomization, even if the subject does not receive the correct treatment, or does not follow the protocol until completion.All randomized patients will be followed and assessed for before and after colonoscopy.

The ITT analysis strategy is defined as follows:

- Is based on an ITT design that aims to collect all outcome data on all randomized subjects;
- Includes a main analysis that keeps subjects in their randomized groups, analyses all available outcome data, and is valid under a named plausible assumption about the missing data;
- Includes sensitivity analyses that consider a range of plausible alternative assumptions about the missing data;
- All randomised individuals are included in sensitivity analyses.
  - 1. **Modified Intention-to-Treat (mITT) Population**

The mITT analysis set included all subjects who took at least one dose of the study drug and attempted colonoscopy after randomization^[25]^. Exclusion criteria include:

- Did not receive any study drug;
- Experienced severe pre-procedure complications (e.g., intestinal obstruction) unrelated to the study intervention;
- Had no post-baseline efficacy data (e.g., no colonoscopy report or missing key endpoints);
- Major protocol deviations affecting primary endpoint assessment (e.g., ineligible patients wrongly randomized);
- Voluntarily withdrew before any efficacy data collection.
  - 1. **Per-protocol (PP) Population**

The per-protocol (PP) population was a further subset of the mITT population, comprising only participants who fully adhered to the study protocol. This included completing the assigned bowel preparation regimen as prescribed and successfully undergoing colonoscopy.

- - 1. **Safety Population**

The safety population includes all patients who received any amount of study drug. Patients who withdraw informed consent immediately after randomization and do not receive any treatment will be excluded from the safety population.Ideally, the safety population will be identical to ITT Population.

- 1. **Analysis of Primary Efficacy Outcome**

For the primary outcome analysis, the proportions of adequate bowel preparation will be compared 3 group arms using modified Poisson regression model. Both adjusted and unadjusted risk ratio and their 95% CIs will be reported. In case of non-convergence of the covariate-adjusted model, the inverse-probability treatment weighting (IPTW) method will be used. We will analyze the primary outcome in the mITT population, and repeat the analysis in the PP population. For shedding cases, follow-up will be performed until the end of the study, and the results will be included in the final analysis. Statistical analysis will be performed on the R version 4.5.1. Details of these are provided in the Statistical Analysis Plan.

- 1. **Analysis of Secondary Efficacy Outcomes**
- Proportion of patients with 0-3 bubble scores (0=None, 3=Severe)(Ordinal data);
- Proportion of patients rating taste as “satisfied” vs. “unsatisfied” (Binary data)；
- Proportion of patients rating educational methods as “satisfied” vs. “unsatisfied” (Binary data)；
- Proportion of patients reporting “better”, “same”, or “worse” sleep vs. Baseline(Categorical data)；
- Proportion of patients with willingness to repeat (yes/no)(Binary data);
- Proportion of patients with compliance (yes/no)(Binary data)；
- Proportion of patients with ≥1 polyp detected (pathology-confirmed)(Binary data);
- Proportion of patients with ≥1 adenoma detected (pathology-confirmed)(Binary data)；
- Proportion of patients with pathology-confirmed cancer(Binary data).

Between-group differences will be tested using the same method as the primary efficacy outcome.The significance of each test is determined at the two-sided alpha level of 0.05. The specific statistical model for analyzing each of these outcome measures will be detailed in the Statistical Analysis Plan (SAP).

- 1. **Analysis of Safety Outcomes**

The safety analysis will be performed on the Safety Population. The proportion of patients experiencing nausea, vomiting, abdominal distension and abdominal pain. The safety population will be identical to mITT Population. The between group difference will be tested using Chi-square test or Fisher’s exact test.

- 1. **Handling of Missing Data**

Every effort will be made to keep missing data, Regular reminders of patient follow-up due dates will be provided to participating centers to facilitate scheduling of follow-up visits. Nevertheless, some missing data may be inevitable due to, for example, loss to follow-up. Since all randomized patients will be included in the primary endpoint analysis, every patient must have a BBPS score. Patients who did not undergo gastroscopy and colonoscopy after taking the medication during the study period will receive the worst scores in all outcome measurements and be included in the analysis.

Missing outcome data of BBPS in the lead analysis will be imputed using multiple imputation. worst-case and best-case analyses will be performed as sensitivity analyses. In the worst-case analysis, all patients with a missing primary endpoint will be considered as a failure (BBPS 0-5) in three treatment groups. In the best-case analysis, all patients with a missing primary endpoint will be considered as a success (BBPS 6-9) in three treatment groups.

Proportions of missing values for all variables will be reported. Variables that will be used to adjust the primary and secondary effect analyses (age, sex, drinking, smoking, diabetes, history of abdominal surgery, first colonoscopy examination) are designated as key variables. Missing values for these variables (if any) will be analyzed for randomness and imputed with standard methods.

- 1. **Covariates and Planned Subgroups**

In the covariates and planned Subgroups-adjusted analysis, the following variables will be adjusted:

- Sex
- Age
- Drinking
- Smoking
- Diabetes
- History of abdominal surgery
- First colonoscopy examination

The aim is to more accurately evaluate the differences in the true efficacy of the three bowel preparation regimens (OSS+Linaclotide group, OSS group, PEG group) by controlling for baseline confounding factors such as demographic characteristics and medical history. In this study, the IPTW method was adopted. The propensity score was calculated based on the multi-class logistic regression model, and adjusted weights were generated to balance the baseline characteristics between groups. By comparing the differences in the success rate of intestinal preparation before and after covariate adjustment, the degree of influence of baseline factors on the evaluation of therapeutic effect can be clarified, thereby more reliably judging the actual effect of different intervention measures. This analytical method helps to reduce confounding bias and improve the internal validity of research results.

1. **Data Safety Monitoring Board**

The Clinical Events Committee will be comprised of three expert physicians independent of the investigational sites. This committee will validate all the complications that occur over the course of the study and categorized for severity and relatedness according to the definition in the AE section. The Clinical Events Committee can request any additional source information and images supporting the AEs to assist with the adjudication.

1. **Ethical and Regulatory Consideration**
   1. **General Requirements and Considerations**

This study followed the ethical principles of the Helsinki Declaration. Approval of the conduct of the trial will be obtained from the Ethics Committees of all participating centers as well as from the local regulatory authorities. The trial will not start in any center before written approval and authorization by the respective Ethics Committee and Regulatory Authority.Any subsequent protocol amendment will be submitted to the Ethics Committee for approval. The involvement of committees in the clinical trial will further ensure that the subjects have the highest priority at any time. The investigators will assure that every patient participating in the trial will receive best medical treatment.

- 1. **Study Monitoring and Quality Control**

The trial design was reviewed by the Steering Committee and approved by Ethics Committees/Regulatory Authorities. Independent Ethics Advisory Board/DSMB approved the protocol and monitor conduct. Committees of experts ensure ethical/safety alerts. Investigators commit to comply with protocol, ICH-GCP, and regulations, providing accurate data. Monitoring team ensures ethical, scientific, and standardized conduct via site visits/calls, assessing protocol compliance, progress, SAE reporting, drug supply, treatment adherence, data quality, etc., and resolving urgent issues.

- 1. **Informed Consent**

Once an eligible patient is confirmed, on-site investigators will discuss the purpose of the trial, procedures, possible benefits, potential risks, and the rights/obligations of participation with the patient or their legal representatives. When all criteria for this study are met, the patient and their family will decide whether or not to participate in this research. The written informed consent must be obtained from all participants in the clinical trial prior to inclusion into the study. Informed consent forms must be written to be easily understood by the participants or their legal representatives, enabling them to understand the purpose of the trial, procedures, possible benefits, potential risks, and the rights/obligations of participation. Randomization will only proceed once informed consent has been signed. Participants have the right to withdraw from the study at any stage of the trial. Each participant must leave contact information to the investigator of the coordinating center. At the same time, the investigator must leave his/her own phone number to the participant so that the participant can find the investigator at any time.

- 1. **Confidentiality**

Personal data will be processed in accordance with Chinese data protection directives and regulations, relevant international legislation and good practices. Data will only be processed for the trial’s purpose. The investigators encode each patient participating in the study by assigning a unique patient identification number to maintain confidentiality standards. This means that all individual patients’ data will be linked to the CRF via a unique identification number throughout the trial. Individual patient medical information will be recorded only in anonymous form. The clinical monitors may inspect source data in order to ensure the accuracy of the data recorded in the CRF.

1. **Administrative Procedures**
   1. **Secrecy Agreement**

The investigators will take all necessary measures to ensure that there is no violation of confidentiality in respect of all information accumulated, acquired or deduced in the course of the trial, other than that information to be disclosed by law.

- 1. **Ownership of Data and Use of the Study Results**

All study-related materials, including unpublished documentation, investigational product information, and electronic/paper case report forms (CRFs), remain the exclusive property of the Sponsor (study initiator). Therefore, the study initiators reserve the right to use the data of the present study, either in the form of CRFs, or in the form of a report, with or without comments and with or without analysis, in order to submit them to the health authorities.

- 1. **Protocol Amendments**

All protocol modifications must be drafted by the investigative team and receive pre-implementation approval from the Research Ethics Board (REB)/Institutional Review Board (IRB), in accordance with local regulatory requirements. Approval must be obtained from the REB/IRB and regulatory authorities (as locally required) before implementation of any changes, except for changes necessary to eliminate an immediate hazard to patients or changes that involve logistical or administrative aspects only.

1. **Data Retention**

The double-reviewed case report form (CRF) will be transmitted to the data management unit for confirmation. The unit supervisor shall verify completeness and endorse the receipt acknowledgment prior to electronic data capture. Following successful database locking, the original CRF documents will be archived at the coordinating research center per ICH-GCP guidelines.

1. **Study Report**

The results of the trial will be reported to the regulatory authorities and ethics committees. The investigators will provide an annual safety report and the final report.

1. **Publications**

According to the pre-defined analysis in the clinical trial protocol, the results of the trial will be published in the appropriate journal (for manuscripts) or meeting (for abstracts).

By signing the clinical trial protocol the investigator agrees that the results of the clinical trial can be used for publication.

The trial will be registered at the Clinical Trial Registry website.

1. **References**

[1] Amitay EL, Niedermaier T, Gies A, et al. Risk Factors of Inadequate Bowel Preparation for Screening Colonoscopy [J]. J Clin Med, 2021,10(12).

[2] Hassan C, East J, Radaelli F, et al. Bowel preparation for colonoscopy: European Society of Gastrointestinal Endoscopy (ESGE) Guideline - Update 2019 [J]. Endoscopy, 2019,51(8): 775-794.

[3] Zhang N, Xu M, Chen X. Establishment of a risk prediction model for bowel preparation failure prior to colonoscopy [J]. BMC Cancer, 2024,24(1): 341.

[4] Mahadeva S. Improving adherence towards bowel preparation for colonoscopy [J]. JGH Open, 2023,7(9): 597-598.

[5] 中国医师协会内镜医师分会消化内镜专业委员会, 中国抗癌协会肿瘤内镜学专业委员会. 中国消化内镜诊疗相关肠道准备指南(2019，上海) %J 中华消化内镜杂志 [J]. 2019, (7): 457-469.

[6] Li CX, Guo Y, Zhu YJ, et al. Comparison of Polyethylene Glycol versus Lactulose Oral Solution for Bowel Preparation prior to Colonoscopy [J]. Gastroenterol Res Pract, 2019,2019: 2651450.

[7] Nam JH, Hong SB, Lim YJ, et al. Comparison of Oral Sulfate Solution and Polyethylene Glycol Plus Ascorbic Acid on the Efficacy of Bowel Preparation [J]. Clin Endosc, 2020,53(5): 568-574.

[8] Theunissen F, Lantinga MA, Ter Borg PCJ, et al. Efficacy of different bowel preparation regimen volumes for colorectal cancer screening and compliance with European Society of Gastrointestinal Endoscopy performance measures [J]. United European Gastroenterol J, 2023,11(5): 448-457.

[9] Cheng P, Chen Q, Li J, et al. 3 liters of polyethylene glycol vs. standard bowel preparation have equal efficacy in a Chinese population: a randomized, controlled trial [J]. Am J Transl Res, 2022,14(8): 5641-5650.

[10] Yan H, Huang H, Yang D, et al. 3 L split-dose polyethylene glycol is superior to 2 L polyethylene glycol in colonoscopic bowel preparation in relatively high-BMI (>/= 24 kg/m(2)) individuals: a multicenter randomized controlled trial [J]. BMC Gastroenterol, 2023,23(1): 427.

[11] He Y, Liu Q, Chen YW, et al. Bowel preparation protocol for hospitalized patients ages 50 years or older: A randomized controlled trial [J]. World J Gastrointest Endosc, 2024,16(1): 18-28.

[12] Othman MF, Zakaria AD, Yahya MM, et al. Comparing Low Volume Versus Conventional Volume of Polyethylene Glycol for Bowel Preparation during Colonoscopy: A Randomised Controlled Trial [J]. Malays J Med Sci, 2023,30(5): 106-115.

[13] Pan P, Zhao S, Wang S, et al. Comparison of the efficacy and safety of an oral sulfate solution and 3-L polyethylene glycol on bowel preparation before colonoscopy: a phase III multicenter randomized controlled trial [J]. Gastrointest Endosc, 2023,98(6): 977-986 e914.

[14] Kim JE. Comments on Oral Sulfate Solution Is as Effective as Polyethylene Glycol with Ascorbic Acid in a Split Method for Bowel Preparation in Patients with Inactive Ulcerative Colitis: A Randomized, Multicenter, and Single-Blind Clinical Trial [J]. Gut Liver, 2024,18(1): 192-193.

[15] Lee HH, Lim CH, Kim JS, et al. Comparison Between an Oral Sulfate Solution and a 2 L of Polyethylene Glycol/Ascorbic Acid as a Split Dose Bowel Preparation for Colonoscopy [J]. J Clin Gastroenterol, 2019,53(10): e431-e437.

[16] Ali IA, Roton D, Madhoun M. Oral sulfate solution versus low-volume polyethylene glycol for bowel preparation: Meta-analysis of randomized controlled trials [J]. Dig Endosc, 2022,34(4): 721-728.

[17] Wang L, Zhang Y, Li J, et al. Efficacy of Polyethylene Glycol Electrolyte Powder Combined With Linaclotide for Colon Cleansing in Patients With Chronic Constipation Undergoing Colonoscopy: A Multicenter, Single-Blinded, Randomized Controlled Trial [J]. Clin Transl Gastroenterol, 2024,15(6): e1.

[18] Song J, Xu Y, Chen C, et al. The Effects of Combined Use of Linaclotide and Polyethylene Glycol Electrolyte Powder in Colonoscopy Preparation for Patients With Chronic Constipation [J]. Surg Laparosc Endosc Percutan Tech, 2024,34(2): 129-135.

[19] Liu WQ, Shu L, Zhou X, et al. Evaluation of the efficacy of polyethylene glycol in combination with different doses of linaclotide in a fractionated bowel preparation for colonoscopy: a prospective randomized controlled study [J]. Int J Colorectal Dis, 2024,39(1): 143.

[20] Sherazi SAA, Goyal H. Impact of 1-day versus 3-day low residue diet for bowel preparation before colonoscopy: more is always not better [J]. Eur J Gastroenterol Hepatol, 2021,33(1S Suppl 1): e1104-e1105.

[21] Hassan C, East J, Radaelli F, et al. Bowel preparation for colonoscopy: European Society of Gastrointestinal Endoscopy (ESGE) Guideline - Update 2019 [J]. Endoscopy, 2019,51(8): 775-794.

[22] Yoon JY, Kim HG, Cho YS, et al. 1 L- versus 2 L-polyethylene glycol with ascorbic acid for bowel preparation in elderly patients: a randomized multicenter study [J]. Surg Endosc, 2022,36(8): 5724-5733.

[23] Lai EJ, Calderwood AH, Doros G, et al. The Boston bowel preparation scale: a valid and reliable instrument for colonoscopy-oriented research [J]. Gastrointest Endosc, 2009,69(3 Pt 2): 620-625.

[24] Calderwood AH, Schroy PC, 3rd, Lieberman DA, et al. Boston Bowel Preparation Scale scores provide a standardized definition of adequate for describing bowel cleanliness [J]. Gastrointest Endosc, 2014,80(2): 269-276.

[25] Arieira C, Dias de Castro F, Boal Carvalho P, et al. Bowel cleansing efficacy for colonoscopy: prospective, randomized comparative study of same-day dosing with 1-L and 2-L PEG + ascorbate [J]. Endosc Int Open, 2021,9(11): E1602-e1610.
