# Supplementary material for: Application of oral sulfate solution combined with linaclotide in bowel preparation for colonoscopy
Source: Front Med (Lausanne). 2026 Mar 10;13:1696298. doi: 10.3389/fmed.2026.1696298 (PMC13014616; doi:10.3389/fmed.2026.1696298)
Supplement: Supplementary file 3 [file Supplementary_file_3.docx]

**Application of Oral Sulfate Solution Combined with Linaclotide in Bowel Preparation for Colonoscopy**

**Statistical Analysis Plan**

Ji Xuan,MD

1 Jinling Clinical Medical College，Nanjing Medical University, Nanjing, China

2 Jinling Hospital, Affiliated Hospital of Medical School, Nanjing University, Nanjing,China

Email：xuanji @nju.edu.cn

**SAP Authors**

Yuxiu Liu, Prof, Data and Statistics Division of Department of Critical Care Medcine, Affiliated Jinling Hospital, Medical School of Nanjing University, Nanjing, China

Mengjie Lu, PhD, Health Science Center, Ningbo University, Ningbo, Zhejiang, China;

| **SAP version history** | | |
| --- | --- | --- |
| Version Date | SAP Version # | Details of Changes |
| **2025/07** | **1.0** |  |

**Contents**

**[1. Abbreviations and Definitions of Terms 1](#_Toc20861)**

**[2. PREFACE 3](#_Toc25825)**

**[3. PURPOSE OF SAP 3](#_Toc16760)**

**[4. VALIDATION OF SAP AND AMENDMENTS 4](#_Toc23838)**

**[5. OBJECTIVES AND ENDPOINTS 4](#_Toc21723)**

**[5.1 Study Objectives 4](#_Toc12486)**

**[5.2 Study Endpoints (Target Variables) 4](#_Toc10765)**

**[5.2.1 Primary Efficacy Endpoint 4](#_Toc21458)**

**[5.2.2 Secondary Efficacy Endpoints 4](#_Toc8101)**

**[5.2.3 Safety Endpoints 5](#_Toc757)**

**[6. STUDY METHODS 5](#_Toc24094)**

**[6.1 Overall Study Design and Plan 6](#_Toc28937)**

**[6.2 Selection of Study Population 7](#_Toc28682)**

**[6.2.1 Inclusion criteria 7](#_Toc7406)**

**[6.2.2 Exclusion criteria 7](#_Toc25848)**

**[6.3 Method of Treatment Assignment and Randomization 7](#_Toc31591)**

**[6.4 Treatment masking (Blinding) 8](#_Toc7271)**

**[7. FORMAL ANALYSES AND REPORTING 8](#_Toc15531)**

**[8. SAMPLE SIZE DETERMINATION 8](#_Toc385)**

**[9. ANALYSIS POPULATIONS 8](#_Toc29646)**

**[9.1 Intention-to-Treat (ITT) Population 9](#_Toc1388)**

**[9.2 Modified intention-to-treat (mITT) population 9](#_Toc22065)**

**[9.3 Safety Population 9](#_Toc21881)**

**[10. GENERAL ISSUES FOR STATISTICAL ANALYSIS 10](#_Toc15245)**

**[10.1 Analysis Software 10](#_Toc29563)**

**[10.2 Methods for Withdrawals and Missing Data 10](#_Toc2916)**

**[10.2.1 Withdrawals 10](#_Toc18944)**

**[10.2.2 Missing data 10](#_Toc27348)**

**[10.3 Data Transformations 11](#_Toc25424)**

**[10.4 Multiple Comparisons and Multiplicity 11](#_Toc22047)**

**[10.5 Covariates and Planned Subgroups 12](#_Toc24920)**

**[10.6 Derived and Computed Variables 12](#_Toc27500)**

**[10.7 Presentation of Results 12](#_Toc1511)**

**[11. PATIENT DISPOSITION 13](#_Toc6276)**

**[12. BASELINE CHARACTERISTICS 13](#_Toc21997)**

**[13. EFFECTIVENESS ANALYSIS 13](#_Toc11172)**

**[13.1 Primary Outcome 13](#_Toc30618)**

**[13.2 Statistical hypothesis 14](#_Toc1937)**

**[13.3 Crude analysis 15](#_Toc10898)**

**[13.4 Covariates adjusted analysis 15](#_Toc32143)**

**[13.5 Sensitivity analysis 16](#_Toc29323)**

**[13.6 Subgroup analysis 17](#_Toc9204)**

**[14. SAFETY ANALYSIS 17](#_Toc4110)**

**[15. TABLES AND FIGURES FOR THE MAIN PAPER 18](#_Toc30316)**

**[16. FUNDINGS 18](#_Toc65)**

**[17. REFERENCES 19](#_Toc13601)**

1. **Abbreviations and Definitions of Terms**

| **Abbreviations /acronyms** | **Definitions** |
| --- | --- |
| ADR | Adenoma Detection Rate |
| AE | Adverse Event |
| BBPS | Boston Bowel Preparation Scale |
| BBS | Bowel Bubble Score |
| BMI | Body Mass Index |
| BP | Blood Pressure |
| CRF | Case Report Form |
| CI | Confidence Interval |
| CSR | Clinical Study Report |
| DSMB | Data Safety Monitoring Board |
| ESGE | European Society of Gastrointestinal Endoscopy |
| GEE | Generalized Estimating Equation |
| GCP | Good Clinical Practice |
| ICH-E9 | International Conference on Harmonization - Statistical principles for Clinical Trials |
| ITT | Intention-to-Treat |
| IPTW | Inverse-Probability Treatment Weighting |
| mITT | Modified Intention-to-Treat |
| OSS | Oral Sulfate Solution |
| PDR | Polyp Detection Rate |
| PEG | Polyethylene Glycol |
| RCT | Randomized Controlled Trial |
| SAP | Statistical Analysis Plan |
| SAE | Serious Adverse Event |
| TEAEs | Treatment-Emergent Adverse Events |

1. **PREFACE**

This Statistical Analysis Plan (SAP) describes the planned analysis and reporting for trial protocol entitled “Efficacy and Safety of Oral Sulfate Solution Combined with Linaclotide for Bowel Preparation Before Colonoscopy: A Randomized Controlled Trial” (https://www.clinicaltrials.gov; Unique identifier: NCT06091735).

This trial is being completed to assess the efficacy and safety of Oral Sulfate Solution (OSS) combined with linaclotide for bowel preparation prior to colonoscopy.

The structure and content of this SAP provides sufficient details to meet the requirements determined by the National Medical Products Administration and International Conference on Harmonization of Technical Requirements for Registration of Pharmaceuticals for Human Use (ICH-E9): Guidance on Statistical Principles in Clinical Trials^[1]^. All work planned and reported for this SAP will follow internationally recognized guidelines, published by the American Statistical Association ^[2,3]^and the Royal Statistical Society, for statistical practice.

In preparing this SAP, the following documents were reviewed:

- Clinical Study Protocol Version.
- Case report forms (CRFs).
- ICH-E9 Guidance on Statistical Principles for Clinical Trials.
- Guidelines for the Content of Statistical Analysis Plans in Clinical Trials.

Readers of this SAP are encouraged to also read the clinical trial protocol to understand the implementation details of this study, the operational aspects of clinical evaluation, and the schedule of patients completing this study.

1. **PURPOSE OF SAP**

The objective of this Statistical Analysis Protocol (SAP) is to delineate the pre-specified statistical evaluations intended to facilitate the compilation of the Clinical Study Report (CSR). All statistical procedures specified within this SAP will constitute the foundational analytical content for regulatory dossiers (including IND/NDA/BLA submissions) and subsequent scholarly publications. Additionally, investigative analyses not explicitly predefined in this SAP may be undertaken to inform strategic decisions in the clinical development program. Any retrospective analyses conducted beyond the scope of this SAP will be distinctly characterized as post-hoc in the corresponding CSR section.

1. **VALIDATION OF SAP AND AMENDMENTS**

The SAP should be validated and signed by:

- Principal Investigator
- Chair of Steering Committee
- Chair of Data Safety and Monitoring Board
- Senior statistician

After validation, the SAP may be amended, and all changes will be clearly tracked. Any amendments can occur only before the database is locked and the treatment code is unblinded.

1. **OBJECTIVES AND ENDPOINTS**
   1. **Study Objectives**

The primary objective is to explore the efficacy and safety of OSS+ Linaclotide in bowel preparation.

- 1. **Study Endpoints (Target Variables)**
     1. **Primary Efficacy Endpoint**

The primary efficacy outcome measure was the bowel cleansing quality, assessed using the Boston Bowel Preparation Scale (BBPS). Total BBPS scores and segment-specific scores (right colon, transverse colon, left colon) were compared among the three groups. Adequate bowel preparation was defined as a total BBPS score ≥6 and segmental scores ≥2 in all colon segments. The adequate rate was calculated as: adequate rate=Adequately prepared cases/Total cases ×100%.

- - 1. **Secondary Efficacy Endpoints**

The secondary efficacy outcomes are as follows:

- Proportion of patients with 0-3 bubble scores (0=None, 3=Severe)(Ordinal data);
- Proportion of patients rating taste as “satisfied” vs. “unsatisfied” (Binary data)；
- Proportion of patients rating educational methods as “satisfied” vs. “unsatisfied” (Binary data)；
- Proportion of patients reporting “better”, “same”, or “worse” sleep vs. Baseline(Categorical data)；
- Proportion of patients with willingness to repeat (yes/no)(Binary data);
- Proportion of patients with compliance (yes/no)(Binary data)；
- Proportion of patients with ≥1 polyp detected (pathology-confirmed)(Binary data);
- Proportion of patients with ≥1 adenoma detected (pathology-confirmed)(Binary data)；
- Proportion of patients with pathology-confirmed cancer(Binary data).
  - 1. **Safety Endpoints**

Safety was evaluated by documenting treatment-emergent adverse events (TEAEs) (nausea, vomiting, abdominal distension, abdominal pain) via a standardized electronic case report form (eCRF).

- The proportion of patients experiencing nausea, vomiting, abdominal distension and abdominal pain.
- Serious adverse events.

1. **STUDY METHODS**

**Patients undergoing colonoscopy**

**Randomization**

**Inclusion and Exclusion Criteria**

**OSS**

**OSS+Linaclotide**

**PEG**

**Outcomes**

**Clinical efficacy**

**Adverse events**

**Evaluation criterion**

**Statistic analysis**

Figure 1. Research flowchart.

- 1. **Overall Study Design and Plan**

This study is a single-center, single-blind, randomized controlled trial (RCT). Patients will be randomly assigned in a 1:1:1 ratio to one of three groups: OSS+Linaclotide group, OSS group, or PEG group. A total of at least 444 patients (148 per group) are planned to be recruited at the Endoscopy Center of Jinling Hospital, Medical School of Nanjing University.After the completion of bowel preparation, colonoscopy was performed to assess the quality of bowel preparation and obtain the primary outcome indicators.The study patient flow outline following is shown in Figure 1.

- 1. **Selection of Study Population**
     1. **Inclusion criteria**
- Age 18-80 years，either sex;
- Individuals scheduled to undergo elective colonoscopy;
- Capable of adhering to the study protocol, follow-up visits, and completion of all study-related assessments (including validated scales).
  - 1. **Exclusion criteria**
- Severe cardiopulmonary, hepatic, or renal dysfunction, including:Severe heart failure (New York Heart Association Class III or IV), recent acute myocardial infarction, or unstable angina;Uncontrolled hypertension (systolic BP ≥160 mmHg or diastolic BP ≥100 mmHg despite antihypertensive therapy); Chronic liver disease, chronic kidney disease, cirrhosis, or ascites;
- Suspected gastrointestinal obstruction or perforation.
- Pregnant or lactating women, or individuals planning pregnancy during the study period;
- Mental illness or physical disability;
- Allergic to bowel preparation drugs;
- Failure to undergo colonoscopy after bowel preparation completion;
- Inability to tolerate diagnostic colonoscopy;
- Voluntary withdrawal;
- Participation in another clinical trial within 3 months prior to enrollment or during the study period.
  1. **Method of Treatment Assignment and Randomization**

Randomization will be done by a web-based APP (Jinlingshu) on mobile phone or computer (https://jinlingshu.com/). The automated system will assign an appropriate set of study medication to each patient. Patients will be randomly assigned in a 1:1:1 ratio to one of three groups: OSS+Linaclotide group, OSS group, or PEG group. Randomization will be stratified by participating centre with permutation block size of 6. Randomization will be completely concealed by having both web-based real-time allocation (All drugs will have a unique number. Subjects will be assigned a random serial number according to the time they were enrolled, and corresponding masked medications will be provided).

- 1. **Treatment masking (Blinding)**

The single-blind method adopted in this study, namely observer blind. The clinical data assessors and collectors will be blinded to treatment assignment.The Data Safety Monitoring Board (DSMB) will have access to masked data.

1. **FORMAL ANALYSES AND REPORTING**

All formal planned analyses identified in the protocol and in this SAP will be performed only after the formal database lock. Before database lock and completion of the final analyses, a blinded data review meeting will be held. Also, no database may be locked, random code unblinded, or analyses completed until this SAP has been approved and signed.

Any post-hoc, exploratory analyses completed to support planned study analyses, which were not identified in this SAP, will be documented and reported in appendices. Any results of these unplanned analyses will also be clearly identified in appendices.

1. **SAMPLE SIZE DETERMINATION**

Drawing on data from previous research, we set the success rates for the OSS+Linaclotide group, the OSS group, and the PEG group at 90%^[4]^, 86%^[5]^, and 69.36%^[6]^, respectively. We set the significance level of the two - tailed test at α = 0.05 and the statistical power at 80%. We allocated samples to each group in a 1:1:1 ratio. The minimum sample size for each group was calculated to be 118 cases. Considering a 20% dropout rate, we plan to include at least 148 subjects in each group.This estimation was performed based on PASS (NCSS, LLC. Kaysville, Utah, USA) version 15.0.

1. **ANALYSIS POPULATIONS**

All primary, secondary effectiveness, and safety endpoints will be analyzed for both the modified intention-to-treat (mITT) and per-protocol (PP) population. Patients who withdraw informed consent immediately after randomization and do not receive any treatment should be excluded from all analysis populations:

The following analysis populations are planned for the studies:

- 1. **Intention-to-Treat (ITT) Population**

The ITT population includes all patients randomized into the trial and who were recorded as receiving any amount of study drug, even if the subject does not receive the correct treatment, or does not follow the protocol until completion^[7]^. The ITT population will be the primary analysis population for the efficacy endpoints and subjects will be analysed according to the treatment group to which they were assigned at randomization^[8]^.

The ITT analysis strategy is defined as follows:

- Is based on an ITT design that aims to collect all outcome data on all randomized subjects;
- Includes a main analysis that keeps subjects in their randomized groups, analyses all available outcome data, and is valid under a named plausible assumption about the missing data;
- Includes sensitivity analyses that consider a range of plausible alternative assumptions about the missing data;
- All randomised individuals are included in sensitivity analyses.
  1. **Modified intention-to-treat (mITT) population**

The mITT analysis set included all subjects who took at least one dose of the study drug and attempted colonoscopy after randomization^[9]^. Exclusion criteria include:

- Did not receive any study drug;
- Experienced severe pre-procedure complications (e.g., intestinal obstruction) unrelated to the study intervention;
- Had no post-baseline efficacy data (e.g., no colonoscopy report or missing key endpoints);
- Major protocol deviations affecting primary endpoint assessment (e.g., ineligible patients wrongly randomized);
- Voluntarily withdrew before any efficacy data collection.
  1. **Per-protocol (PP) Population**

The per-protocol (PP) population was a further subset of the mITT population, comprising only participants who fully adhered to the study protocol. This included completing the assigned bowel preparation regimen as prescribed and successfully undergoing colonoscopy.

- 1. **Safety Population**

The safety population includes all patients who received any amount of study drug. Patients who withdraw informed consent immediately after randomization and do not receive any treatment will be excluded from the safety population.Ideally, the safety population will be identical to mITT Population.

1. **GENERAL ISSUES FOR STATISTICAL ANALYSIS**
   1. **Analysis Software**

All statistical analyses will be performed using SPSS or R version 4.5.1. Figures were drawn using GraphPad Prism.

- 1. **Methods for Withdrawals and Missing Data**
     1. **Withdrawals**

If any subject withdraws from the study prematurely, they are required to complete the withdrawal visit in the case report form. The reasons for withdrawal will be listed in a summary table. Subjects who withdrew before the subject’s last follow up will be included in the analysis.

- - 1. **Missing data**

The handling of missing data will follow the principles specified in the ICH-E9 and the CPMP/EWP/1776/99Rev1^[1]^. Guideline on Missing Data in confirmatory trials Guidelines^[10]^.To minimize missing data, particularly for drug evaluation endpoints pre-colonoscopy, a comprehensive follow-up protocol will be implemented. After the patients are randomized, the patients or their families and doctors will keep WeChat (one of the most used Chinese social networking software similar to Facebook and WhatsApp), home address, and phone numbers of the patient's family in person to avoid loss to follow-up.

- - - 1. **Baseline covariates**

Missing baseline covariates will be addressed through simple imputation methods in the covariate-adjusted analysis, provided that the proportion of subjects with missing values for each covariate is less than 5%. For continuous variables, missing values will be imputed using random values drawn from a normal distribution, with the mean and standard deviation derived from the available data. For categorical variables, missing values will be imputed using random values from a uniform distribution, with probabilities P1, P2, ..., and Pk estimated from the sample.

If the proportion of subjects with missing values for a covariate is ≥5% then the missing values will be imputed using Markov chain Monte Carlo (MCMC) methods using SAS PROC MI. A total of 10 multiply imputed dataset will be generated. The seed for the imputations will be 120.

- - - 1. **Efficacy outcomes**

Sensitivity analyses based on different hypotheses about the missingness pattern of the primary outcome will be performed to test for the robustness of the primary analysis results. Primary analysis of the primary outcome will be conducted in the complete case of the mITT population (in which patients with missing data will be excluded).

- 1. **Data Transformations**

Most primary/secondary endpoints are categorical, requiring no data transformation.While continuous variables may undergo transformation to meet distributional assumptions for parametric analyses, nonparametric statistical methods (e.g., Wilcoxon rank-sum, Kruskal-Wallis tests) will serve as the primary analytical approach. Any decision to transform specific variables will be made exclusively during the blinded review phase, prior to treatment code unblinding, and will be guided by formal diagnostic criteria for distributional normality.

- 1. **Multiple Comparisons and Multiplicity**

To address potential type I error inflation in this three-arm randomized trial comparing OSS+Linaclotide, OSS alone, and PEG, we implemented a predefined multiple comparison adjustment strategy. Primary analyses commenced with omnibus testing using one-way ANOVA for normally distributed continuous variables or Kruskal-Wallis tests for non-normally distributed data, with significance defined as p<0.05. For significant omnibus results, subsequent pairwise comparisons employed Bonferroni correction (adjusted α=0.0167 for three comparisons). All multiplicity adjustment methods were prospectively specified in the statistical analysis plan prior to unblinding and database lock, in accordance with ICH E9 guidelines. Secondary endpoints were analyzed using similar multiplicity controls where appropriate, while safety analyses were performed without adjustment to maintain sensitivity for adverse event detection.

- 1. **Covariates and Planned Subgroups**

In the covariates and planned Subgroups-adjusted analysis, the following variables will be adjusted:

- Sex
- Age
- Drinking
- Smoking
- History of abdominal surgery
- First colonoscopy examination

The aim is to more accurately evaluate the differences in the true efficacy of the three bowel preparation regimens (OSS+Linaclotide group, OSS group, PEG group) by controlling for baseline confounding factors such as demographic characteristics and medical history. In this study, the Inverse Probability Processing weighting (IPTW) method was adopted. The propensity score was calculated based on the multi-class logistic regression model, and adjusted weights were generated to balance the baseline characteristics between groups. By comparing the differences in the success rate of intestinal preparation before and after covariate adjustment, the degree of influence of baseline factors on the evaluation of therapeutic effect can be clarified, thereby more reliably judging the actual effect of different intervention measures. This analytical method helps to reduce confounding bias and improve the internal validity of research results.

- 1. **Derived and Computed Variables**

Some derived and computed variables have been initially identified and described as appropriate later in the document. The SAP will not be amended for additional variables that are not related to the primary target or secondary target variables.

- 1. **Presentation of Results**

Description of continuous variables includes the mean±standard deviation, or median and interquartile range. Description of categorical variables includes the frequency and percentage n (%). Description of ordered variables includes the frequency, percentage and cumulative percentage.

1. **PATIENT DISPOSITION**

The number of patients for each of the following categories will be summarized.

- Randomized patients
- Patients completing the study and not completing the study (grouped by treatment and main reason)
- Patients included in the ITT population
- Patients included in the mITT population
- Patients included in the PP population
- Patients included in the safety population

1. **BASELINE CHARACTERISTICS**

Patient demographics and baseline clinical characteristics will be summarized descriptively by treatment group. The following demographic information will be listed and summarized for subjects in each treatment group: age, and sex, BMI and others. Baseline characteristics include: medical history(drinking, smoking, hypertension, diabetes, history of abdominal surgery, history of polyp resection, constipation and others),First colonoscopy examination, indication of colonoscopy, endoscopist. The summaries will be provided for the ITT and mITT populations.

The continuous data will be presented as mean and standard deviation, or median and interquartile range as appropriate; categorical data will be presented as n (%).

1. **EFFECTIVENESS ANALYSIS**

All efficacy analyses will provide the point estimate of treatment effect with associated two sided 95% CI. Statistical analyses of the primary and secondary efficacy endpoints will primarily be based on the mITT population. Secondarily, the same analyses will be repeated on the PP population.Assessment of efficacy will be conducted based on BBPS.

- 1. **Primary Outcome**

The BBPS is a standardized tool for assessing the quality of bowel preparation before colonoscopy, developed by the Boston University Medical Center^[11]^. This scale divides the colon into three parts (the right colon, the transverse colon, and the left colon), and scores each part separately, with a total score range of 0 to 9 points^[12]^.

- 0 points: The mucosa is invisible and cannot be evaluated due to solid feces or liquid covering it.
- 1 point: The mucosal part is visible, but there is residual feces/turbid fluid that affects observation (rinsing or aspiration is required).
- 2 points: A small amount of residual feces/liquid, with clearly visible mucous membranes (slight impact, no additional cleaning required).
- 3 points: The mucosa is completely visible, with no residual feces or fluid.

The primary efficacy outcome measure for this study is:Adequate rate of bowel preparation.Adequate bowel preparation was defined as a total BBPS score ≥6 and segmental scores ≥2 in all colon segments. The adequate rate was calculated as: Adequate rate=Adequately prepared cases/Total cases ×100%.We will retain the video footage and images of the patient's colonoscopy. The colonoscopy physician will first conduct a BBPS score after the colonoscopy is completed, and then two researchers will review it.Disagreements will be resolved by consensus. The electronic Data Capture System (EDC) was programmed with allocation concealment to maintain blinding of assessors, and participants were explicitly instructed to withhold any treatment-related information that could compromise study blinding.

The primary hypothesis to be tested is that the effect of bowel preparation after taking intestinal cleansers. The primary effectiveness analysis for this trial is The therapeutic effect differences between the OSS+Linaclotide group and the OSS group as well as the PEG group.

- 1. **Statistical hypothesis**

For this three-arm randomized controlled trial comparing bowel preparation efficacy among OSS+Linaclotide, OSS alone, and PEG groups, we established the following statistical hypotheses:

- **Primary Hypothesis (Omnibus Test):**

H₀: There is no difference in adequate bowel preparation rates among the three groups (π₁ = π₂ = π₃)

H₁: At least one group differs in adequate bowel preparation rates

- **Pairwise Comparisons (If omnibus test significant):**

1. **OSS+Linaclotide vs. PEG:**

H₀: π₁ = π₃

H₁: π₁ ≠ π₃

1. **OSS+Linaclotide vs. OSS :**

H₀: π₁ = π₂

H₁: π₁ ≠ π₂

1. **OSS vs. PEG:**

H₀: π₂ = π₃

H₁: π₂ ≠ π₃

- 1. **Crude analysis**

The treatment effect will be estimated using modified Poisson regression model with the primary outcome as the dependent variable and treatment group as an independent variable to determine the risk ratio (RR) and its corresponding 95% CI.

In case of nonconvergence of this model, the following generalized linear models (GLMs) will be fitted sequentially until a model is converged：

- GLM with Binomial distribution and log link function;
- GLM with Negative Binomial distribution and log link function;
- GLM with Binomial distribution and logit link function, from which odds ratio (OR) will be converted into RR^[13,14]^.

In addition, risk difference will be estimated using a GLM (identity-binomial regression) as supportive measurement of treatment effect.

- 1. **Covariates adjusted analysis**

Modified Poisson regression model adjusted for sex, age, drinking, smoking, diabetes, history of abdominal surgery, first colonoscopy examination will also be used to calculate the adjusted RR with 95% CI. Imputation for baseline missing covariates will be made for covariate adjusted analysis.

The model may not converge when all covariates are introduced into the modified Poisson model simultaneously. If non-convergence occurs, IPTW analysis will be performed to calculate the adjusted RR with 95% CI^[15,16]^.

The main conclusion will be drawn from the covariates-adjusted analysis of the primary outcome on the ITT population.

- 1. **Sensitivity analysis**

To assess the impact of missing data on the primary endpoint (compliance rate of bowel preparation) on the estimation of treatment effects and verify the robustness of the results.

The therapeutic effect was re-estimated in the following preset scenarios using the same statistical model as the main analysis (modified Poisson regression regression) :

- **Worst-case scenario:**

All subjects with missing primary endpoints were considered to have failed bowel preparation (such as BBPS < 6 points or incomplete colonoscopy).

- **Best-case scenario:**

All subjects with missing primary endpoints were considered to have successful bowel preparation (e.g., BBPS ≥ 6 points).

- **Multiple imputation:**

Based on baseline covariates (such as age and history of defecation) and observed outcome data, 20 imputed datasets were generated and the results were combined for analysis.

- **Assessment of center effect:**

A generalized estimating equation (GEE) model with Poisson distribution and log link function will be fitted to estimate the adjusted RR between the intervention and control treatment with treatment, adjusted for sex, age, drinking, smoking, diabetes, history of abdominal surgery, first colonoscopy examination as a cluster effect. If the covariates adjusted GEE Poisson model does not converge, IPTW method will be used.

- 1. **Subgroup analysis**

Exploratory subgroup analyses of the primary endpoint will be conducted across predefined subgroups using modified Poisson regression. For each subgroup category, the relative risk (RR) and corresponding 95% confidence interval (CI) will be estimated. Results will be visually summarized in forest plots to evaluate potential treatment effect modification by subgroup variables.

- Sex (male vs. female)
- Age (<50 vs ≥50 years )
- Drinking (yes vs no)
- Smoking (yes vs no)
- History of abdominal surgery (yes vs no)
- First colonoscopy examination (yes vs no)

Treatment effect modification by subgroup variables will be evaluated using a modified Poisson regression model, incorporating treatment assignment, subgroup indicator, and their interaction term as covariates. The statistical significance of effect heterogeneity will be determined by the P-value associated with the interaction term.

Subgroup analysis will be performed on three ITT and mITT populations.

1. **SAFETY ANALYSIS**

- **Adverse Events**

AE frequencies and incidences will be stratified by treatment group and categorized using the Medical Dictionary for Regulatory Activities (MedDRA) System Organ Class (SOC). Data collection will continue through the colonoscopy examination day or final study contact, whichever occurs first.

The number and percentage of subjects experiencing adverse events will be summarized by preferred term. Results will be presented for each of the following 3 groups:

1. OSS+ Linaclotide group;
2. OSS group;
3. PEG group;

The incidence of subjects with ≥1 SAEs will be summarized by preferred term using absolute counts and percentages (n, %).

All AE analyses will utilize data adjudicated by the Clinical Events Committee (CEC) or Data Safety Monitoring Board (DSMB), which supersedes investigator-reported information.

1. **TABLES AND FIGURES FOR THE MAIN PAPER**

The proposed table and figures for the main results are described below:

Table 1 will report the key baseline characteristics of participants by patient group.

Table 2 will report the primary outcomes.

The summary of secondary outcomes will be presented in a supplementary table.

Figure 1 will be the diagram of bowel preparation methods.

Figure 2 will report a proportional bar chart of the BBPS distribution in each treatment group.

Figure 3 will report forest plots of the primary outcome for pre-specified subgroup.

Figure 4 will report the incidence of adverse reactions in each group.

The research flowchart will be presented in a supplementary figure.

All tables and pictures except the main text should be included in the supplementary file.

1. **FUNDINGS**

This study received financial support from the Jinling Hospital, Affiliated Hospital of Medical School, Nanjing University (2023LCYYQH024 and 2023LCYYXH013). All authors are very appreciative of the financial support.

1. **REFERENCES**

[1] Kahan BC, Hindley J, Edwards M, et al. The estimands framework: a primer on the ICH E9(R1) addendum [J]. Bmj, 2024,384: e076316.

[2] Homer V, Yap C, Bond S, et al. Early phase clinical trials extension to guidelines for the content of statistical analysis plans [J]. Bmj, 2022,376: e068177.

[3] Gamble C, Krishan A, Stocken D, et al. Guidelines for the Content of Statistical Analysis Plans in Clinical Trials [J]. Jama, 2017,318(23): 2337-2343.

[4] Hassan C, East J, Radaelli F, et al. Bowel preparation for colonoscopy: European Society of Gastrointestinal Endoscopy (ESGE) Guideline - Update 2019 [J]. Endoscopy, 2019,51(8): 775-794.

[5] Lee HH, Lim CH, Kim JS, et al. Comparison Between an Oral Sulfate Solution and a 2 L of Polyethylene Glycol/Ascorbic Acid as a Split Dose Bowel Preparation for Colonoscopy [J]. J Clin Gastroenterol, 2019,53(10): e431-e437.

[6] Yoon JY, Kim HG, Cho YS, et al. 1 L- versus 2 L-polyethylene glycol with ascorbic acid for bowel preparation in elderly patients: a randomized multicenter study [J]. Surg Endosc, 2022,36(8): 5724-5733.

[7] White IR, Horton NJ, Carpenter J, et al. Strategy for intention to treat analysis in randomised trials with missing outcome data [J]. Bmj, 2011,342: d40.

[8] Molero-Calafell J, Burón A, Castells X, et al. Intention to treat and per protocol analyses: differences and similarities [J]. J Clin Epidemiol, 2024,173: 111457.

[9] Arieira C, Dias de Castro F, Boal Carvalho P, et al. Bowel cleansing efficacy for colonoscopy: prospective, randomized comparative study of same-day dosing with 1-L and 2-L PEG + ascorbate [J]. Endosc Int Open, 2021,9(11): E1602-e1610.

[10] Mehrotra DV, Hemmings RJ, Russek-Cohen E. Seeking harmony: estimands and sensitivity analyses for confirmatory clinical trials [J]. Clin Trials, 2016,13(4): 456-458.

[11] Lai EJ, Calderwood AH, Doros G, et al. The Boston bowel preparation scale: a valid and reliable instrument for colonoscopy-oriented research [J]. Gastrointest Endosc, 2009,69(3 Pt 2): 620-625.

[12] Calderwood AH, Schroy PC, 3rd, Lieberman DA, et al. Boston Bowel Preparation Scale scores provide a standardized definition of adequate for describing bowel cleanliness [J]. Gastrointest Endosc, 2014,80(2): 269-276.

[13] Morris TP, Walker AS, Williamson EJ, et al. Planning a method for covariate adjustment in individually randomised trials: a practical guide [J]. Trials, 2022,23(1): 328.

[14] Austin PC, Stuart EA. Moving towards best practice when using inverse probability of treatment weighting (IPTW) using the propensity score to estimate causal treatment effects in observational studies [J]. Stat Med, 2015,34(28): 3661-3679.

[15] Zou GY, Donner A. Extension of the modified Poisson regression model to prospective studies with correlated binary data [J]. Stat Methods Med Res, 2013,22(6): 661-670.

[16] Zou G. A modified poisson regression approach to prospective studies with binary data [J]. Am J Epidemiol, 2004,159(7): 702-706.
